# Cannabidiol Enhances Microglial Beta-Amyloid Peptide Phagocytosis and Clearance via Vanilloid Family Type 2 Channel Activation

**DOI:** 10.3390/ijms23105367

**Published:** 2022-05-11

**Authors:** Shaobin Yang, Yaqin Du, Xiaoqian Zhao, Qi Tang, Wei Su, Yuemeng Hu, Peng Yu

**Affiliations:** College of Life Sciences, Northwest Normal University, Lanzhou 730070, China; 15682820645@163.com (Y.D.); zxq990726@163.com (X.Z.); 18893724560@163.com (Q.T.); suwei21@mails.ucas.ac.cn (W.S.); 17393154495@163.com (Y.H.)

**Keywords:** cannabidiol, phagocytosis, TRPV2, autophagy, Aβ1-42

## Abstract

Alzheimer’s disease (AD) is associated with the accumulation and aggregation of amyloid in the brain. The cation channel TRPV2 may mediate the pathological changes in mild cognitive impairment. A high-affinity agonist of TRPV2 named cannabidiol is one of the candidate drugs for AD. However, the molecular mechanism of cannabidiol via TRPV2 in AD remains unknown. The present study investigated whether cannabidiol enhances the phagocytosis and clearance of microglial Aβ via the TRPV2 channel. We used a human dataset, mouse primary neuron and microglia cultures, and AD model mice to evaluate TRPV2 expression and the ability of microglial amyloid-β phagocytosis in vivo and in vitro. The results revealed that TRPV2 expression was reduced in the cortex and hippocampus of AD model mice and AD patients. Cannabidiol enhanced microglial amyloid-β phagocytosis through TRPV2 activation, which increased the mRNA expression of the phagocytosis-related receptors, but knockdown of TRPV2 or Trem2 rescued the expression. TRPV2-mediated effects were also dependent on PDK1/Akt signaling, a pathway in which autophagy was indispensable. Furthermore, cannabidiol treatment successfully attenuated neuroinflammation while simultaneously improving mitochondrial function and ATP production via TRPV2 activation. Therefore, TRPV2 is proposed as a potential therapeutic target in AD, while CBD is a promising drug candidate for AD.

## 1. Introduction

Alzheimer’s disease (AD) is the most common form of dementia, which is accompanied by progressive cognitive impairment in the elderly [1,2]. AD has, so far, affected over 50 million individuals, and this number is expected to reach 150 million in the next 40 years [3]. The coformation of amyloid plaques remains the most accepted etiology of AD and is associated with the accumulation and aggregation of amyloid-β (Aβ) in the brain, followed by changes in brain function, such as tau-mediated neuronal injury, metabolism, neuroinflammation, synaptic impairment, and neuronal death, eventually leading to structural changes in the brain [4,5,6]. Therefore, inhibiting the synthesis, accumulation, and aggregation of Aβ remains the central strategy for the prevention of AD [7]. However, a few trials targeting Aβ have failed to exhibit promising clinical outcomes because of the side effects and signal targets, such as verubecestat (BACE-1 inhibitor) and avagacestat (gamma-secretase inhibitor) [8,9]. Most efforts in this regard have led to the discovery of drugs that promote Aβ clearance in the preclinical stage or mild cognitive impairment (MCI). Microglia are the primary immune cells of the central nervous system (CNS), which may assist in preventing Aβ accumulation via phagocytosis-mediated clearance [10]. Increasing evidence suggests that inducing receptor expression on myeloid cells (Trem2) has an important effect on microglial Aβ phagocytosis and AD pathology [11,12]. Sodium rutin reportedly promotes Aβ clearance by enhancing the expression levels of phagocytosis-related receptors in microglia. Microglia phagocytic dysfunction caused by Trem2 KO could, therefore, be eventually rescued through sodium rutin treatment [13]. Interestingly, the genetic analysis of peripheral blood from human patients has revealed that several genes are associated with the risk of developing AD. This set of genes includes transient receptor potential vanilloid 2 (TRPV2), which reportedly mediates the pathological changes in MCI, as revealed in a previous weighted gene coexpression network analysis (WGCNA) [14]. The TRPV2 channel is expressed in the peripheral and CNS, mainly related to its ability to permeate Ca^2+^ [15,16]. TRPV2 is reported to act as a thermosensor for noxious heat in the neurons of the dorsal root and trigeminal ganglia. TRPV2 is also suggested to be involved in developing axon outgrowth and various osmo-related or mechanical sensations [17]. TRPV2 reportedly plays an indispensable role in the maintenance of cardiac structure and function via insulin-like growth factor-1 (IGF-1) receptor/phosphoinositide 3-kinase (PI3K)/protein kinase B (Akt) signaling pathway [18]. The TRPV2 ion channel is also critical for the phagocytosis of macrophages and microglia [19]. Despite these reports, the role of TRPV2 in AD has not been studied well so far. However, a high-affinity agonist of the TRPV2 named cannabidiol (CBD) is a candidate drug for AD, which acts via cannabinoid receptors [20,21,22]. CBD is a natural component extracted from *Cannabis sativa*. It exhibits various properties, including neuroprotective and anti-inflammatory properties [23,24]. However, the molecular mechanism underlying the action of CBD via TRPV2 in AD remains unknown so far.

Autophagy is reported to have an important role in AD pathology, as suggested by an increasing number of reports. Intracellular Aβ may induce autophagy, while a deficiency of autophagy increases extracellular Aβ plaques [25,26]. In the AD mouse model and cells, microglia were demonstrated to engulf and degrade neuron-released Aβ through selective autophagy [27]. In addition, the CBD-induced TRPV2-dependent autophagic process induces glioma stemlike cells in a PI3K/Akt signaling-dependent manner [28]. Recent transcriptomic and transmission electron microscopy analyses conducted in the APP/PS1 mouse model of AD have suggested that CBD upregulates autophagy [29]. However, the relationship between CBD-regulated Aβ clearance via TRPV2 and autophagy remains to be elucidated to date.

Therefore, the present study was aimed to investigate whether CBD enhances microglial Aβ phagocytosis and clearance via the TRPV2 channel. A human dataset was used along with cellular and animal models to demonstrate that CBD improved Aβ phagocytosis and clearance through TRPV2 activation in microglia, accompanied by autophagy and changes in energy metabolism. The results of the present study demonstrated that TRPV2 is a potential therapeutic target in AD, while CBD is a promising drug candidate for AD. 

## 2. Results

### 2.1. TRPV2 Levels Were Reduced in AD Patients and AD Mice

**Figure 1:**
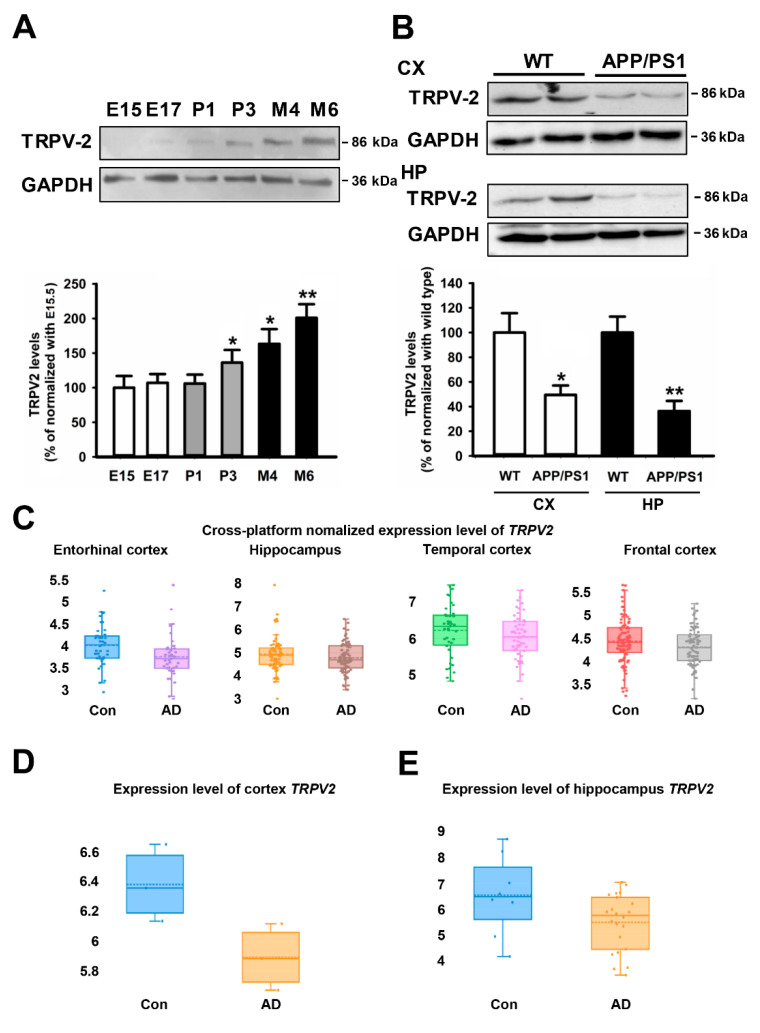
TRPV2 levels were decreased in both AD patients and APP/PS1 mice. (**A**) The TRPV2 levels were measured in the brain whole-protein extracts from mice at the indicated age (E is embryos, P is days and M is months). The data are represented as the mean ± SEM (*n* = 4 mice/group). * *p* < 0.05, and ** *p* < 0.01 are compared to the E15.5 by the Tukey’s test. (**B**) Cortex and hippocampus protein extracts were obtained at 6 months of age from wild-type (WT) and two transgenic AD mice models (APP/PS1) who were subjected to the TRPV2 antibody. The data are represented as the mean ± SEM for four different mice per genotype. * *p* < 0.05, and ** *p* < 0.01 compared to controls as obtained by the Student’s *t*-test. (**C**) Transcriptional expression levels of TRPV2 in entorhinal, temporal, and frontal cortex and hippocampus tissues of the patients with AD in the cross-platform database (entorhinal cortex: GSE26927, GSE26972, GSE48350, GSE5281; hippocampus: GSE28146, GSE29378, GSE36980, GSE48350, GSE5281; temporal cortex: GSE29652, GSE36980, GSE37263, GSE5281; frontal cortex: GSE12685, GSE36980, GSE48350, GSE5281, GSE53890, GSE66333; control, *n* = 19; AD patient, *n* = 19). (**D**) Transcriptional downregulation of TRPV2 in the entorhinal cortex of the tissue samples from patients with AD in the GSE26972 database (control, *n* = 19; AD patient, *n* = 19). (**E**) Transcriptional downregulation of TRPV2 in the hippocampus of the tissue samples from patients with AD in the GSE28146 database (control, *n* = 19; AD patient, *n* = 19).

First, the TRPV2 expression in the brains of wild-type mice was determined between the E15.5 stage and 6 months to determine the effect of TRPV2 on AD development. The results revealed that the TRPV2 protein expression increased progressively with aging (Figure 1A, F(5, 32) = 7.475, P3: 100 ± 16.67% vs. 138.67 ± 18.21%, *p* = 0.024; M4: 100 ± 16.67% vs. 158.26 ± 23.56%, *p* = 0.012; M6: 100 ± 16.67% vs. 203.42 ± 16.49%, *p* = 0.001). As depicted in Figure 1B, at the age of 6 months, the levels of TRPV2 protein in the cortex and hippocampus of the APP/PS1 mouse brain were significantly reduced compared with the levels in the wild-type (WT) littermates (value: CX: 100 ± 18.11% vs. 48.58 ± 8.87%, t(6) = 2.916; *p* = 0.027; HP: 100 ± 18.11% vs. 36.21 ± 7.52%, t(6) = 4.432; *p* = 0.004). In order to further assess the involvement of TRPV2 in AD, the changes in the expression of TRPV2 were analyzed using the AlzData web server (www.alzdata.org accessed on 27 July 2018). The cross-platform normalized expression of TRPV2 was different in the cortex and hippocampus. In the GSE26972 dataset, human TRPV2 was significantly reduced in the entorhinal cortex samples of AD patients compared with the control patients (*p* = 0.035, Figure 1C), with no changes in the hippocampus, temporal cortex, and frontal cortex (all *p >* 0.05, Figure 1C). In the GSE28146 dataset, as depicted in Figure 1E, only hippocampal TRPV2 levels were decreased in the samples of AD patients compared with control patients (*p* = 0.017). 

When characterizing the expression pattern of TRPV2 in the brain, the TRPV2 protein expression in microglia was observed to be significantly increased compared with the neuronal expression of this protein, as revealed in the immunoblot analysis (see Supplementary Materials for Figure S1: value: 100 ± 11.21% vs. 8.78 ± 0.87%, t(6) = 11.564; *p* < 0.001). Together, these results indicated that TRPV2 levels were reduced in AD patients and AD mice and that microglial TRPV2 could play an important role in the development of AD. 

### 2.2. CBD Enhances Microglial Aβ Phagocytosis via TRPV2 Channels

**Figure 2:**
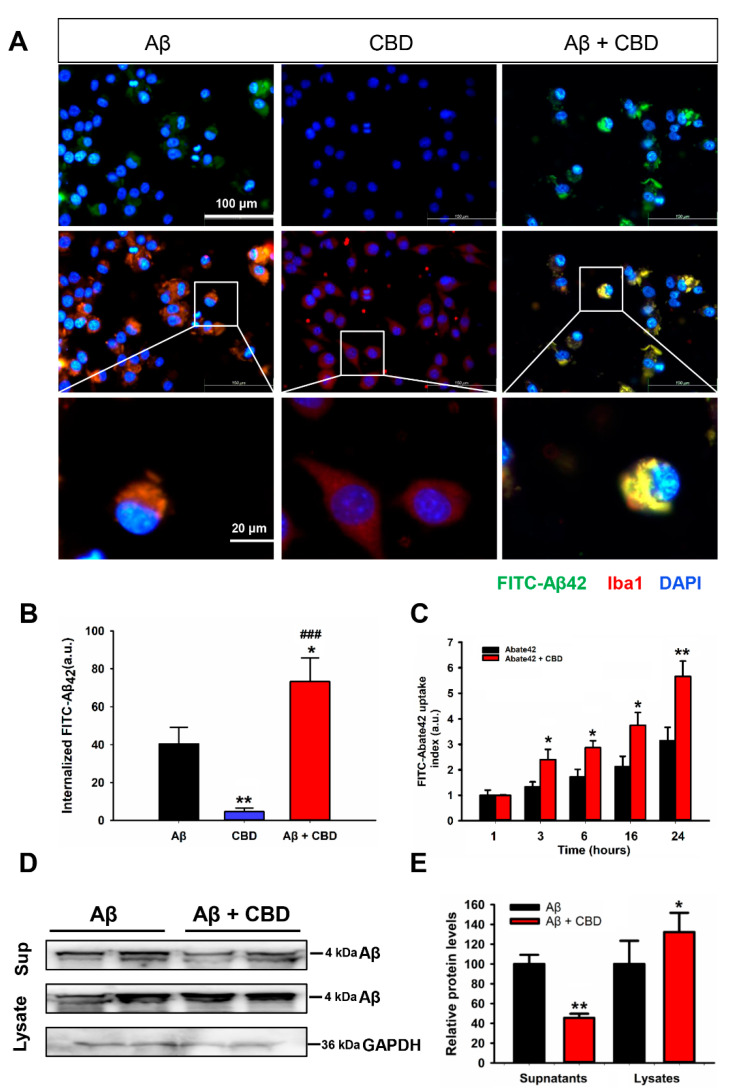
CBD enhanced microglial Aβ phagocytosis via the TRPV2 channels. (**A**) Primary microglia cells were plated at a density of 5 × 10^4^ in poly-D-lysine-coated wells of 24-well plates containing 10% FBS DMEM medium. After 24 h of treatment with CBD (5 μM), FITC-Aβ42 (1 μg/mL) was added to the medium. Immunofluorescence analysis of the microglial phagocytosis of FITC-Aβ42 was performed after allowing uptake for 4 h in microglia cells. (**B**) Quantification of the internalized FITC-Aβ42 using ImageJ software (*n* = 36 to 42 per group). a.u., arbitrary units from 3/4 individual mice. (**C**) FITC-Aβ42 uptake index in the presence or absence of CBD after uptake for the indicated time in microglia cells, calculated based on the phagocytosis assay. (**D**) Microglial Aβ42 uptake was analyzed using Western blotting after 4 h of incubation with Aβ42 oligomer in the presence or absence of CBD. (**E**) Quantification of the protein levels using Image J software (*n* = 4 per group). * *p* < 0.05 and ** *p* < 0.01 are compared with the Aβ by the Student’s *t*-test; ^###^ *p* < 0.001 between Aβ and CBD + Aβ by Student’s *t*-test. The scale bars = 20 μm.

Aβ accumulation and aggregation are the main reasons underlying the pathogenesis of AD [6]. Extracellular Aβ is phagocytosed mainly by the microglia present in the brain [10]. In order to determine whether TRPV2 accelerated Aβ phagocytosis in microglia, cells were treated with fluorescein isothiocyanate (FITC)-labeled Aβ, and the engulfment of these fluorescent peptides was analyzed. CBD treatment significantly increased the efficiency of FITC-Aβ uptake by microglia cells compared with CBD or Aβ alone (Figure 2A,B, F(2, 9) = 35.622, Aβ vs. CBD: 40.35 ± 8.81% vs. 4.56 ± 1.97%, *p* = 0.004; Aβ vs. CBD + Aβ: 40.35 ± 8.81% vs.73.32 ± 12.40%, *p* = 0.011; Aβ vs. CBD + Aβ: 40.35 ± 8.81% vs. 73.32 ± 12.40%, *p* < 0.001). Consistent with this, CBD improved the increase in the fluorescence intensity of FITC-Aβ in a time-dependent manner (Figure 2C: 3 h: Aβ vs. Aβ + CBD: 1.32 ± 0.24% vs. 2.56 ± 0.47%, t(10) = −2.721; *p* = 0.022; 6 h: Aβ vs. Aβ + CBD: 1.82 ± 0.34% vs. 2.96 ± 0.17%, t(10) = −2.828; *p* = 0.018; 16 h: Aβ vs. Aβ + CBD: 2.12 ± 0.44% vs. 3.76 ± 0.47%, t(10) = −3.003; *p* = 0.013; 24 h: Aβ vs. Aβ + CBD: 3.12 ± 0.56% vs. 5.46 ± 0.59%, t(10) = −3.841; *p* = 0.003). In order to further demonstrate that CBD could promote microglial Aβ phagocytosis, intracellular Aβ and remaining Aβ were analyzed using immunoblot analysis, and the results are presented in Figure 2D,E (value: Sup: Aβ vs. Aβ + CBD: 100 ± 24.65% vs. 42.86 ± 4.27%, t(6) = 5.365; *p* = 0.002; Lys: Aβ vs. Aβ + CBD: 100 ± 23.34% vs. 127.96 ± 21.17%, t(6) = −2.87; *p* = 0.028). CBD could enhance the levels of intracellular Aβ while decreasing the Aβ levels in the supernatant after the Aβ treatment of microglia cells. In order to exclude the possibility that CBD improved the engulfment of Aβ by microglia as CBD could activate TRPV1 and TRPV4, the expression changes were analyzed using the AlzData web server (www.alzdata.org, accessed on 27 July 2018). The cross-platform normalized expressions of both TRPV1 and TRPV4 were not significantly different in the cortex and hippocampus (all *p* > 0.05), while the expression of TRPV4 was not detected in the microglia (Figure S2). Together, these results demonstrated that CBD treatment improved the phagocytic capacity of microglial cells via the TRPV2 channel and promotion of Aβ clearance in the microglia cells.

### 2.3. TRPV2-Mediated Phagocytosis in Microglia Is Attenuated via Inhibition with PDK, Akt, or PERK Antagonists

**Figure 3:**
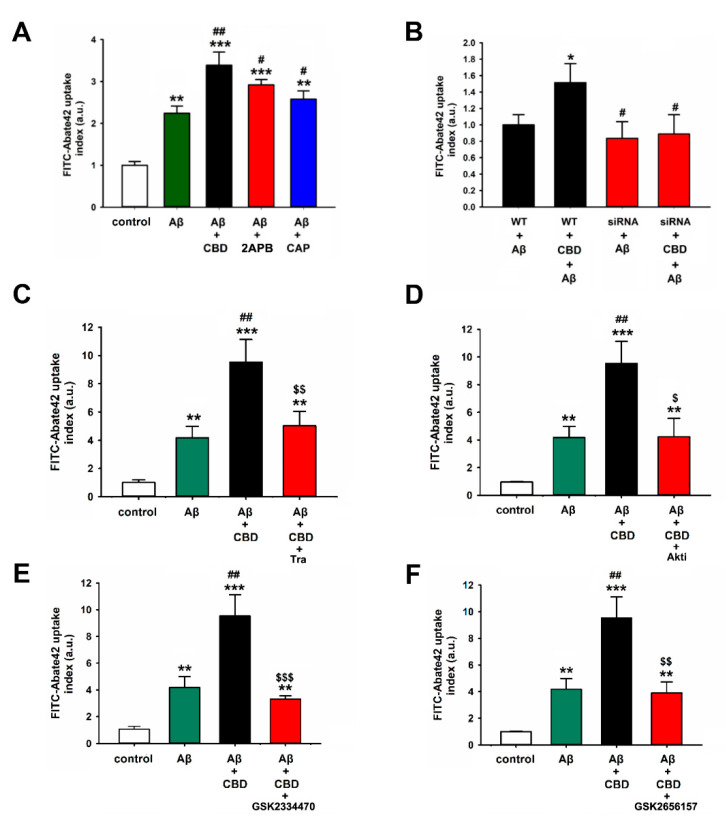
The TRPV2-mediated phagocytosis in microglia cells was attenuated by inhibiting PDK, Akt, or PERK. FITC-Aβ42 uptake index in BV2 microglia cells was analyzed using the phagocytosis assay. (**A**) The presence of CBD (5 μM), 2APB (250 μM), and CAP (capsaicin: 2 μM) after 24 h (*n* = 6). ** *p* < 0.01 and *** *p* < 0.001 are compared with the control by Tukey’s test, and ^#^ *p* < 0.05 and ^##^ *p* < 0.01 are compared with Aβ by Tukey’s test. (**B**) BV2 microglia with knocked-down TRPV2 treated with or without CBD after 24 h (*n* = 4). * *p* < 0.05 is between Aβ and CBD + Aβ by Student’s *t*-test; ^#^ *p* < 0.05 is between Aβ and CBD + Aβ upon siRNA by Student’s *t*-test. (**C**–**F**) BV2 microglia preadded with Tra (tranilast, 75 μM), akti (Akt inhibitor, 10 μM), PDK1 inhibitor (GSK2334470, 1 μM), and PERK inhibitor (GSK2656157, 1 μM) and treated with or without CBD after 24 h (*n* = 5). ** *p* < 0.01 and *** *p* < 0.001 are compared with the control by Tukey’s test; ^##^ *p* < 0.01 is between Aβ and CBD + Aβ by Student’s *t*-test; ^$^ *p* < 0.05, ^$$^ *p* < 0.01, and ^$$$^ *p* < 0.001 are between CBD + Aβ and CBD + Aβ upon inhibitors by Student’s *t*-test.

In order to determine whether the other TRPV2 agonist or TRPV1 mediated microglial phagocytosis, 2-APB was applied to the TRPV2 agonist or capsaicin was applied to the TRPV1 agonist. The uptake assays revealed that Aβ42 and 2-APB or capsaicin-cotreated microglia cells exhibited significantly increased fluorescence intensity compared with the cells treated with Aβ42, although the highest fluorescence intensity of FITC-Aβ42 was exhibited by the cells cotreated with CBD and Aβ (Figure 3A: F(4, 25) = 23.7922, Aβ vs. CBD + Aβ: 2.25 ± 0.18% vs. 3.32 ± 0.37%, *p* = 0.002; Aβ vs. 2-PAB + Aβ: 2.25 ± 0.18% vs. 2.82 ± 0.21%, *p* = 0.013; Aβ vs. 2-PAB + Aβ: 2.25 ± 0.18% vs. 2.63 ± 0.27%, *p* = 0.045). In order to further investigate how CBD affected the TRPV2-mediated enhancement of Aβ phagocytosis, the TRPV2 siRNA and antagonist were used. As expected, the downregulation of TRPV2 significantly reduced the fluorescence intensity in the CBD and Aβ cotreatment group compared with the wild-type group (Figure 3B: F(3, 20) = 5.118, WT + CBD + Aβ vs. siRNA + CBD + Aβ: 1.48 ± 0.39% vs. 0.82 ± 0.37%, *p* = 0.026). Consistent with this, the TRPV2 antagonist tranilast markedly inhibited the effects of the CBD-associated phagocytosis of FITC-Aβ (Figure 3C: F(3, 20) = 26.223, CBD + Aβ vs. tra + CBD + Aβ: 9.53 ± 1.59% vs. 5.02 ± 1.02%, *p* = 0.001). However, tra failed to compromise cell viability in both CBD + Aβ and tra + CBD + Aβ groups (Figure S3A). TRPV2 plays an indispensable role in the maintenance of cardiac structure and function via insulin-like growth factor-1 (IGF-1) receptor/phosphoinositide 3-kinase (PI3K)/protein kinase B (Akt) signaling pathway. PDK1 and PERK signaling pathways are downstream of the PI3K signaling pathway [18]. Next, it was determined whether CBD regulated the TRPV2-mediated Aβ phagocytosis in microglia via the PDK1- and PERK-dependent pathways. As depicted in Figure 3D–F (F(3, 20) = 16.863, CBD + Aβ vs. akti + CBD + Aβ: 9.53 ± 1.59% vs. 4.24 ± 1.32%, *p* = 0.01; F(3, 20) = 38.978, CBD + Aβ vs. GSK2334470 + CBD + Aβ: 9.53 ± 1.59% vs. 3.3 ± 0.24%, *p* < 0.001; F(3, 20) = 25.018, CBD + Aβ vs. GSK2656157 + CBD + Aβ: 9.53 ± 1.59% vs. 3.90 ± 0.8%, *p* = 0.002), CBD enhanced Aβ phagocytosis, which was completely blocked upon treatment with PDK1, Akt, or PERK inhibitors. Together, these results strongly suggest that CBD regulates the TRPV2-mediated Aβ phagocytosis in microglia through the PDK1/Akt and PERK-dependent signaling pathways.

### 2.4. CBD Increased the Expression of Microglial Phagocytic Receptors through TRPV2 Activation

**Figure 4:**
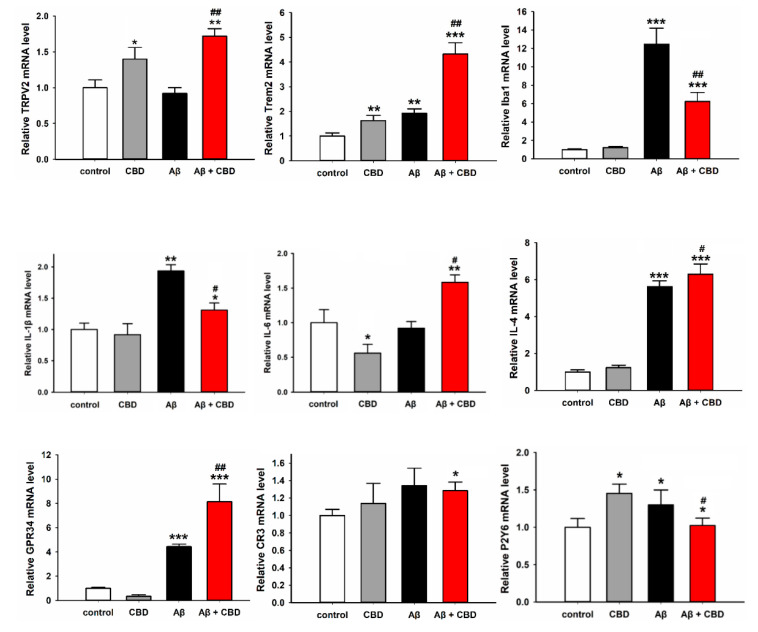
Relative mRNA levels of microglial phagocytic receptors and inflammation factors in the microglia cells treated with or without CBD (5 μM). Primary microglial cells were treated with or without CBD after 12 h, and the mRNA levels were determined through qPCR. The data are represented as the mean ± SEM (*n* = 6). * *p* < 0.05, ** *p* < 0.01, and *** *p* < 0.001 are compared with the control by Tukey’s test; ^#^ *p* < 0.05 and ^##^ *p* < 0.01 are between Aβ and CBD + Aβ by Student’s *t*-test.

In order to explore how CBD modulates microglial phagocytic capacity and inflammation, a quantitative real-time polymerase chain reaction (qRT-PCR) was performed using the RNA from microglia to detect the mRNA expression levels of the presumptive microglial phagocytic receptors, such as Trem2, G protein-coupled receptor 34 (GPR34), complement receptor 3 (CR3), and pyrimidinergic receptor P2Y6 (P2Y6), certain inflammatory factors, proinflammatory cytokines IL-1β and IL-6, and TNFα. As depicted in Figure 4, CBD was observed to increase the levels of the mRNA expression of TRPV2 and Trem2 (TRPV2: F(3, 20) = 12.335, 1 ± 0.11% vs. 1.43 ± 0.21%, *p* = 0.013; Trem2: F(3, 20) = 67.094, 1 ± 0.13% vs. 1.73 ± 0.23%, *p* = 0.008), while Aβ upregulated the mRNA expression levels of Trem2, Iba1, IL-1β, IL-4, and GPR34 when compared with the control group (Trem2: F(3, 20) = 67.094, 1 ± 0.13% vs. 1.93 ± 0.18%, *p* = 0.005; Iba1: F(3, 20) = 56.861, 1 ± 0.08% vs. 12.37 ± 2.18%, *p* < 0.001; IL-1β: F(3, 20) = 8.753, 1 ± 0.07% vs. 1.88 ± 0.17%, *p* = 0.001; IL-4: F(3, 20) = 57.448, 1 ± 0.06% vs. 5.61 ± 0.21%, *p* < 0.001; GPR34: 1 ± 0.08% vs. 4.28 ± 0.13%, *p* < 0.001). Furthermore, cotreatment with CBD and Aβ significantly increased the levels of mRNA expression of TRPV2, Trem2, IL-6, IL-4, and GPR34 compared with the Aβ group (TRPV2: F(3, 20) = 12.335, 0.88 ± 0.07% vs. 1.73 ± 0.21%, *p* = 0.001; Trem2: F(3, 20) = 67.094, 1.93 ± 0.18% vs. 4.23 ± 0.39%, *p* = 0.001; IL-6: F(3, 20) = 16.541, 0.86 ± 0.06% vs. 1.67 ± 0.19%, *p* = 0.012; IL-4: F(3, 20) = 57.448, 5.61 ± 0.21% vs. 6.17 ± 0.45%, *p* = 0.02; GPR34: F(3, 20) = 64.956, 4.28 ± 0.13% vs. 8.15 ± 1.12%, *p* = 0.002). On the contrary, cotreatment with CBD and Aβ markedly reduced the mRNA expression levels of Iba1 and IL-1β compared with the Aβ group (Iba1: F(3, 20) = 56.861, 12.37 ± 2.18% vs. 6.17 ± 0.56%, *p* = 0.002; IL-1β: F(3, 20) = 8.753, 1.88 ± 0.17% vs. 1.28 ± 0.13%, *p* = 0.04). 

**Figure 5:**
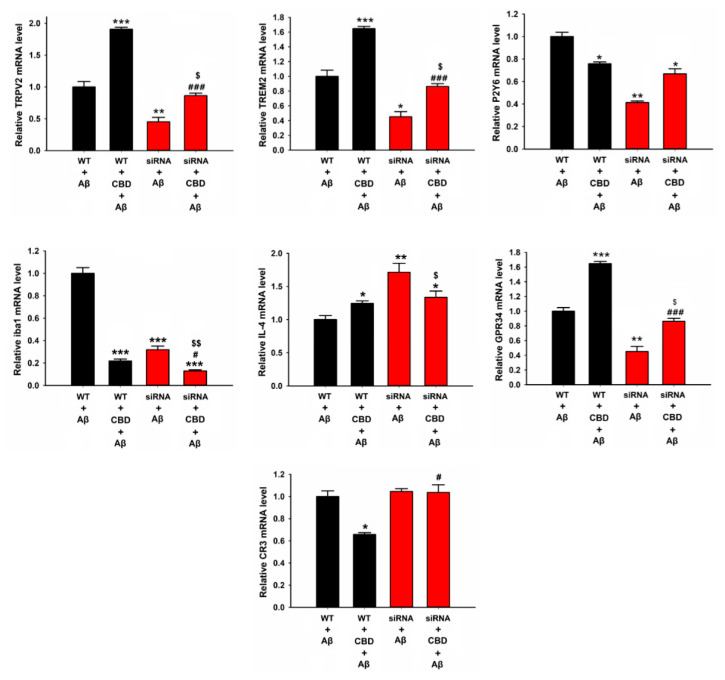
Relative mRNA levels of microglial phagocytic receptors and inflammation factors in microglia cells after knockdown of TRPV2. BV2 microglia were treated with or without CBD (5 μM) after 12 h, and mRNA levels were determined by qPCR. The data are represented as the mean ± SEM (*n* = 4). * *p* < 0.05, ** *p* < 0.01, and *** *p* < 0.001 are compared with wild type treated with Aβ by Tukey’s test; ^#^ *p* < 0.05 and ^###^ *p* < 0.001 mean CBD + Aβ with or without siRNA by Student’s *t*-test. ^$^ *p* < 0.05 and ^$$^ *p* < 0.01 mean CBD knockdown of TRPV2 with or without CBD by Student’s *t*-test.

In order to validate the above results, the expression levels were measured using the TRPV2 siRNA and Trem2 siRNA. As expected, the siRNA treatment did not induce cell death in the microglia (Figure S3B). Furthermore, treatment with CBD remarkably enhanced the mRNA expression of TRPV2, Trem2, GPR34, and IL-4, while it reduced the mRNA expression of P2Y6, Iba1, and CR3 (Figure 5). The mRNA expression levels of TRPV2, Trem2, Iba1, and GPR34 in the Aβ-treated group of TRPV2 knockdown microglia were significantly decreased compared with those in the wild-type control group, while no such change was observed for the mRNA expression levels of IL-4 and CR3 (Figure 5, TRPV2: F(3, 20) = 56.861, WT + CBD + Aβ vs. siRNA + CBD + Aβ: 1.91 ± 0.09% vs. 0.81 ± 0.1%, *p* < 0.001; Trem2: F(3, 20) = 53.818, WT + CBD + Aβ vs. siRNA + CBD + Aβ: 1.63 ± 0.06% vs. 0.83 ± 0.07%, *p* < 0.001; Iba1: F(3, 20) = 193.709, WT + CBD + Aβ vs. siRNA + CBD + Aβ: 0.21 ± 0.01% vs. 0.13 ± 0.01%, *p* = 0.03; GPR34: F(3, 20) = 45.404, WT + CBD + Aβ vs. siRNA + CBD + Aβ: 1.64 ± 0.06% vs. 0.86 ± 0.03%, *p* < 0.001). Similarly, cotreatment with CBD and Aβ of TRPV2 knockdown microglia impaired the upregulation of Iba1 and IL-4 mRNA expression compared with cotreatment with Aβ and siRNA (Figure 5, Iba1: F(3, 20) = 193.709, siRNA + Aβ vs. siRNA + CBD + Aβ: 0.31 ± 0.03% vs. 0.13 ± 0.01%, *p* = 0.01; IL-4: siRNA + Aβ vs. siRNA + CBD + Aβ: F(3, 20) = 12.221, 1.71 ± 0.02% vs. 1.26 ± 0.03%, *p* = 0.039).

**Figure 6:**
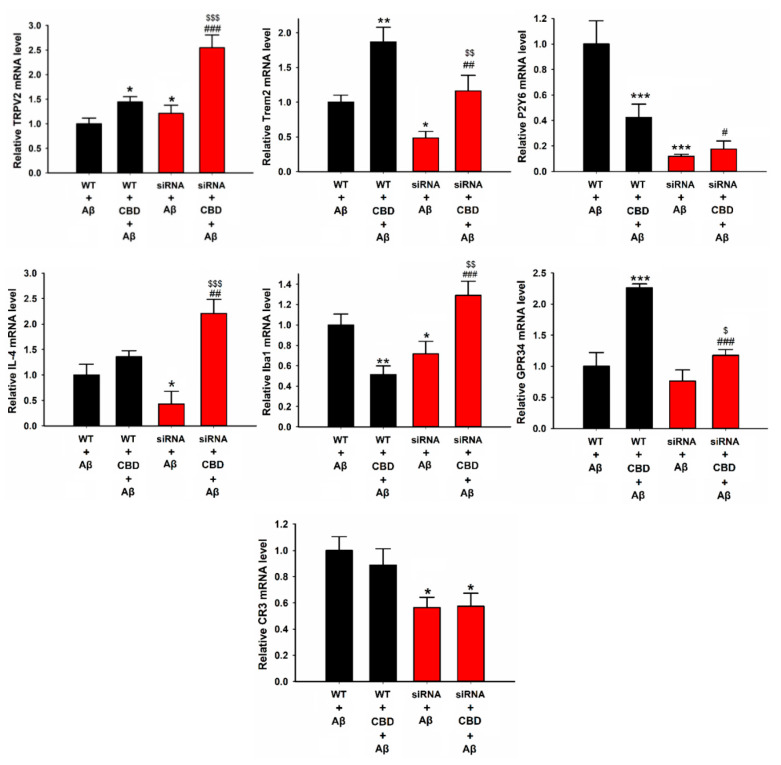
Relative mRNA levels of microglial phagocytic receptors and inflammation factors in microglia cells after knockdown of Trem2. BV2 microglia were treated with or without CBD (5 μM) after 12 h, and mRNA levels were determined by qPCR. The data are represented as the mean ± SEM (*n* = 4). * *p* < 0.05, ** *p* < 0.01, and *** *p* < 0.001 are compared with wild type treated with Aβ (1 μg/mL) by Tukey’s test; ^#^ *p* < 0.05, ^##^ *p* < 0.01, and ^###^ *p* < 0.001 mean CBD + Aβ with or without siRNA by Student’s *t*-test. ^$^ *p* < 0.05, ^$$^ *p* < 0.01, and ^$$$^ *p* < 0.001 mean CBD knockdown of Trem2 with or without CBD by Student’s *t*-test.

The next step was to determine how Trem2 knockdown affected these transcription expression levels in mouse microglia in response to CBD and Aβ. As depicted in Figure 6, the Trem2 knockdown group exhibited markedly reduced mRNA expressions of Trem2, P2Y6, IL-4, Iba1, and CR3 compared with the wild-type group after treatment with Aβ, while the levels of TRPV2 and GPR34 were not altered (Figure 6, Trem2: F(3, 8) = 33.831, WT + Aβ vs. siRNA + Aβ: 1.0 ± 0.1% vs. 0.48 ± 0.09%, *p* = 0.024; P2Y6: F(3, 8) = 51.478, WT + Aβ vs. siRNA + Aβ: 1.0 ± 0.18% vs. 0.11 ± 0.05%, *p* < 0.001; Iba1: F(3, 8) = 27.530, WT + Aβ vs. siRNA + Aβ: 1.0 ± 0.10% vs. 0.71 ± 0.12%, *p* = 0.022; CR3: F(3, 8) = 3.754, WT + Aβ vs. siRNA + Aβ: 1.0 ± 0.1% vs. 0.56 ± 0.27%, *p* = 0.025). Similarly, upon cotreatment with CBD and Aβ, the mRNA expressions of Trem2, P2Y6, and GRP34 were decreased in Trem2-deficient microglia compared with wild-type group cells, while the levels of TRPV2, IL-4, and Iba1 were significantly increased (Figure 6, TRPV2: F(3, 8) = 49.106, WT + CBD + Aβ vs. siRNA + CBD + Aβ: 1.44 ± 0.1% vs. 2.54 ± 0.26%, *p* < 0.001; Trem2: F(3, 8) = 33.831, WT + CBD + Aβ vs. siRNA + CBD + Aβ: 1.86 ± 0.21% vs. 1.16 ± 0.22%, *p* = 0.004; P2Y6: F(3, 8) = 51.478, WT + CBD + Aβ vs. siRNA + CBD + Aβ: 0.42 ± 0.1% vs. 0.17 ± 0.06%, *p* =0.025; Iba1: F(3, 8) = 27.530, WT + CBD + Aβ vs. siRNA + CBD + Aβ: 0.51 ± 0.08% vs. 1.28 ± 0.14%, *p* < 0.001; GPR34: F(3, 8) = 55.54, WT + CBD + Aβ vs. siRNA + CBD + Aβ: 2.26 ± 0.06% vs. 1.17 ± 0.09%, *p* < 0.001; IL-4: WT + CBD + Aβ vs. siRNA + CBD + Aβ: 1.36 ± 0.11% vs. 2.2 ± 0.27%, *p* < 0.007). Surprisingly, the expressions of TRPV2, Trem2, IL-4, Iba1, and GPR34 in Trem2-deficient Aβ microglia were ameliorated upon CBD treatment. Together, these results suggested that microglial phagocytic receptors are dispensable for the CBD-mediated enhancement of Aβ phagocytosis via the TRPV2 channel.

### 2.5. CBD Induced Autophagy in Microglial Cells by Promoting TRPV2 and Upregulating Akt

**Figure 7:**
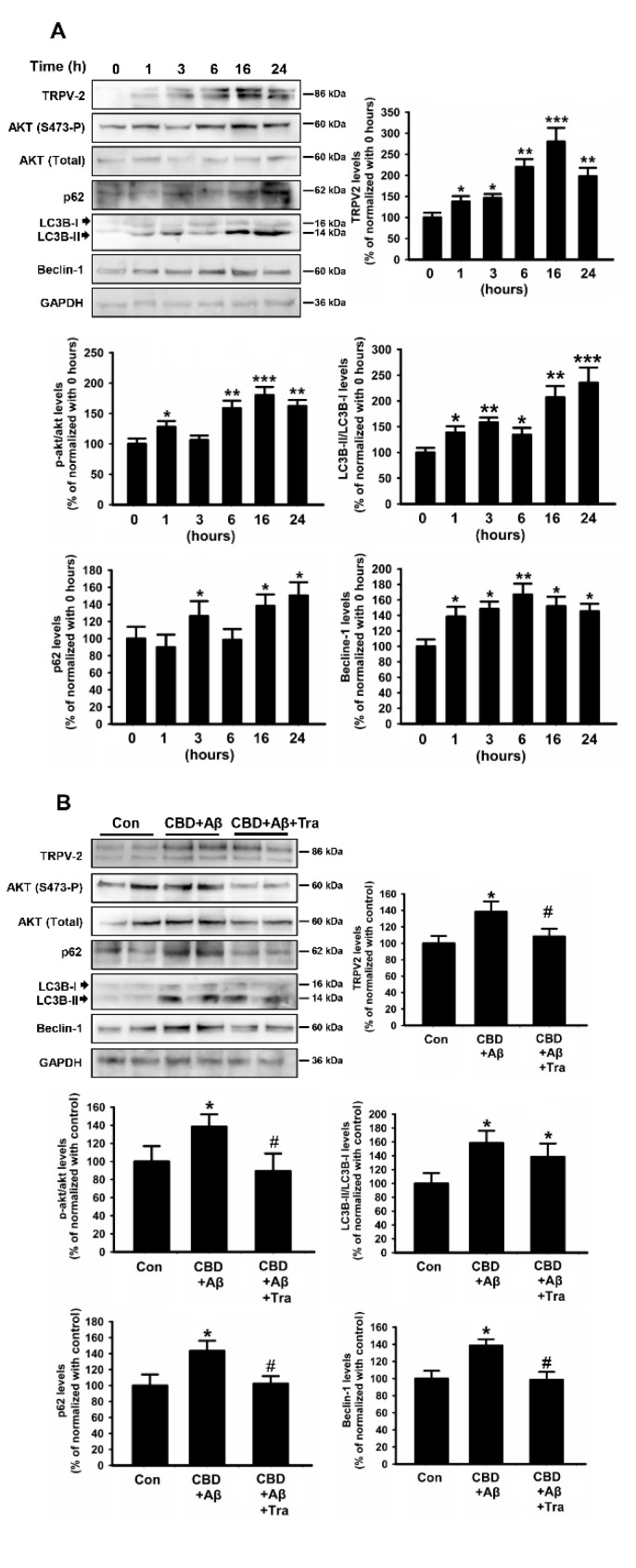
CBD induced autophagy in microglial cells via TRPV2 by promoting the upregulation of Akt. (**A**) BV2 microglia cells incubated with Aβ42 were analyzed using Western blot in the presence of CBD (5 μM) for the indicated time. (**B**) BV2 microglial cells were pretreated with Tra (tranilast 75 μM) for 1 h and then treated with CBD for 24 h. Band densitometry quantification of TRPV2. The autophagy flux expression was normalized to GAPDH. The phosphorylation of Akt was normalized to the total Akt level (*n* = 4 per group). * *p* < 0.05, ** *p* < 0.01, and *** *p* < 0.001 are compared with the control by Tukey’s test. ^#^ *p* < 0.05 mean CBD + Aβ with or without siRNA by Student’s *t*-test.

Aβ clearance is one of the promising strategies for AD prevention and treatment via autophagy [30]. PI3K/Akt signaling plays an important role in the maintenance of TRPV2 function [31]. Mechanistically, the PDK1 or Akt inhibitor reportedly inhibited the TRPV2 protein expression in microglia, and IGF-1 treatment significantly increased TRPV2 expression and the phosphorylation of Akt at Ser473 in mouse N2a neuroblastoma cells (Figure S4A, Akti, TRPV2: F(2, 9) = 52.467, 100 ± 13.27% vs. 28.67 ± 6.41%, *p* = 0.001; pAkt: F(2, 9) = 38.195, 100 ± 11.25% vs. 52.34 ± 12.31%, *p* = 0.003; PDK1 inhibitor, TRPV2: F(2, 9) = 52.467, 100 ± 13.27% vs. 9.28 ± 5.31%, *p* < 0.001; pAkt: F(2, 9) = 38.195, 100 ± 11.25% vs. 11.32 ± 6.65%, *p* < 0.001 and B, TRPV2: 100 ± 9.37% vs. 145.27 ± 16.51%, t (6) = −3.458; *p* = 0.013; pAkt: 100 ± 9.57% vs. 132.24 ± 10.21%, t(6) = −2.911; *p* = 0.027). After confirming the relationship between TRPV2 and the Akt pathway in microglia cells, the next step was to determine whether TRPV2-mediated Aβ phagocytosis and clearance relied on Akt signaling-regulated autophagy. The results revealed that CBD improved the TRPV2 protein expression and the phosphorylation of Akt in microglia cells in a time-dependent manner, with the highest expression induced at 16 h in the presence of Aβ (Figure 7A, TRPV2: F(5, 18) = 37.405, 100 ± 11.37% vs. 268.67 ± 26.51%, *p* < 0.001; pAkt: F(5, 18) = 26.784, 100 ± 9.35% vs. 172.34 ± 17.41%, *p* < 0.001). In order to assess the effect of CBD on autophagy flux, the levels of autophagy-related proteins LC3B-II, p62, and Beclin-1 were determined. Interestingly, CBD upregulated the levels of these proteins in microglia cells in a time-dependent manner, with the highest upregulation of LC3B-II and p62 occurring at 24 h and that of Beclin-1 observed at 6 h after treatment (Figure 7A, LC3B-II: F(5, 18) = 18.745, 100 ± 10.27% vs. 228.27 ± 32.81%, *p* < 0.001; p62: F(5, 18) = 12.929, 100 ± 12.65% vs. 145.94 ± 18.31%, *p* = 0.01; Beclin-1: F(5, 18) = 17.626, 100 ± 11.65% vs. 165.34 ± 15.81%, *p* = 0.006). Consistent with this, the fluorescence intensity of p62 was significantly increased at 24 h after co-treatment with CBD and Aβ compared with treatment with Aβ alone (Figure S5).

Furthermore, the autophagy mechanism in microglia cells was confirmed by the inhibition of TRPV2 activation using tranilast. As depicted in Figure 7B, the expression of TRPV2 and the phosphorylation of Akt at Ser473 induced by Aβ and CBD together were significantly ameliorated upon treatment with tranilast (Figure 7B, TRPV2: F(2, 9) = 7.59, 138.15 ± 12.35% vs. 104 ± 8.27%, *p* = 0.047; pAkt: F(2, 9) = 11.461, 132.36 ± 7.89% vs. 87.23 ± 15.37%, *p* = 0.011). Similarly, the protein levels of p62 and Beclin-1 enhanced by CBD and Aβ were inhibited from tranilast (Figure 7B, p62: F(2, 9) = 12.617, 145.23 ± 11.78% vs. 102.78 ± 8.91%, *p* = 0.018; Beclin-1: F(2, 9) = 14.512, 141 ± 7.75% vs. 94.64 ± 9.87%, *p* = 0.011). Together, these results suggested that Akt-regulated autophagy is indispensable for TRPV2-mediated Aβ phagocytosis and clearance.

### 2.6. CBD Improved Energy Metabolism via Mitochondrial Functions in Microglial Cells in Response to Aβ

**Figure 8:**
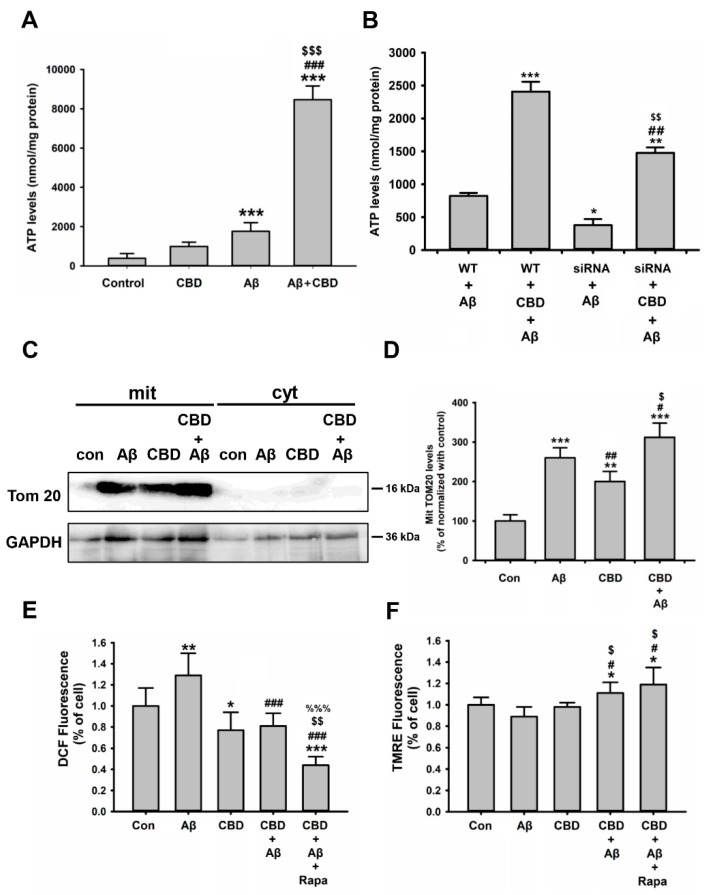
CBD improved energy metabolism via mitochondrial functions in microglia cells. (**A**) Total ATP production in BV2 microglia under different conditions as indicated. Aβ (1 μg/mL) and/or CBD (5 μM) were added to the cells for 24 h prior to measurement (*n* = 6 from three independent experiments). The data are represented as the mean ± SEM. * *p* < 0.05 and *** *p* < 0.001 are compared with the controls by Tukey’s test; ^###^ *p* < 0.001 means CBD + Aβ and CBD by Student’s *t*-test; ^$$^ *p* < 0.01 and ^$$$^ *p* < 0.001 is CBD with or without Aβ by Student’s *t*-test. (**B**) Total ATP production in WT and TRPV2 knockdown microglia cells treated with or without CBD for 24 h (*n* = 6 from three independent experiments). * *p* < 0.05, ** *p* < 0.01, and *** *p* < 0.001 compared with the controls by Tukey’s test; ^##^ *p* < 0.01 is between Aβ and CBD + Aβ by Student’s *t*-test; ^$$^ *p* < 0.01 is between Aβ and CBD + Aβ upon siRNA by Student’s *t*-test. (**C**) BV2 microglial cells were analyzed for Tom 20 expression in mit (mitochondria) and cyt (cytoplasm) by performing immunoblot analysis under different conditions as indicated using the mitochondrial extraction kit. (**D**) Band densitometry quantification of TRPV2 was normalized to GAPDH (*n* = 4 per group). ** *p* < 0.01 and *** *p* < 0.001 are compared with control by Tukey’s test; ^#^ *p* < 0.05 and ^##^ *p* < 0.01 are compared with Aβ by Tukey’s test; ^$^ *p* < 0.05 is between Aβ and CBD + Aβ by Student’s *t*-test. (**E**) ROS production and (**F**) MMP of BV2 microglial cells under different conditions as indicated. After 1 h of treatment with Rapa (rapamycin, 10 μM), Aβ and/or CBD were added to the cells for 24 h prior to measurement (*n* = 12 from three independent experiments). The fluorescence intensities of DCF and TMRE were quantified and expressed as a percentage relative to the controls. The data are represented as the mean ± SEM. * *p* < 0.05, ** *p* < 0.01, and *** *p* < 0.001 are compared with the controls by Tukey’s test; ^###^ *p* < 0.001 are compared with Aβ by Tukey’s test; ^$$^ *p* < 0.01 is CBD with or without Aβ and Rapa by Student’s *t*-test; ^%%%^ *p* < 0.001 is Aβ with or without CBD and Rapa by Student’s *t*-test.

The metabolic health of microglial cells and their ability to produce ATP appear to be crucial for the effective functioning of these cells [11,32]. Therefore, the next step was to determine whether CBD regulates the energy metabolism of microglia cells treated with Aβ via the TRPV2 channel. As depicted in Figure 8A, ATP production increased significantly in the microglia cells under Aβ treatment (Figure 8A, F(3, 20) = 1612.792, 284.76 ± 50.26% vs. 1789 ± 187.27%, *p* < 0.001) and was further elevated upon cotreatment with CBD and Aβ compared with treatment with Aβ or CBD alone (Figure 8A, F(3, 20) = 1612.792, Aβ: 1789 ± 187.27% vs. 8243 ± 426.79%, *p* < 0.001; CBD: 616.04 ± 47.25% vs. 8243 ± 426.79%, *p* < 0.001). The ATP levels were also markedly increased in the BV2 microglia cells co-cultured with N2a cells under cotreatment with Aβ and CBD compared with the control (Figure S6A), while the ATP levels in N2a cells were not altered upon these treatments (Figure S6B). In order to confirm the relationship between ATP production and TRPV2-mediated microglial Aβ phagocytosis, the TRPV2 siRNA was utilized to inhibit the TRPV2 channel. The results revealed that TRPV2 knockdown markedly reduced the ATP levels induced by Aβ in the microglia cells compared with the wild-type treatment (Figure 8B, F(3, 20) = 42.464, WT + Aβ vs. siRNA + Aβ: 821.87 ± 45.69% vs. 377.91 ± 92.91%, *p* = 0.033). Although CBD treatment rescued the energy metabolism deficit in TRPV2-deficient microglia, it was nonetheless significantly reduced compared with the wild-type treatment (Figure 8B, F(3, 20) = 42.464, WT + CBD + Aβ vs. siRNA + CBD + Aβ: 2408.14 ± 250.61% vs. 1477.51 ± 102.50%, *p* = 0.006; siRNA + Aβ: vs. siRNA + CBD + Aβ: 377.91 ± 92.91% vs. 1477.51 ± 102.50%, *p* = 0.003). 

In order to investigate how CBD enhanced microglial energy metabolism, the outer membrane translocase (Tom) 20 immunoblots, reactive oxygen species (ROS) generation, and mitochondrial membrane potential (MMP) were analyzed. First, the mitochondrial extraction kit and Western blot analysis were utilized to evaluate Tom 20 levels in microglial cells. The expression levels of the Tom 20 protein were mainly localized to mitochondria and increased statistically upon Aβ and/or CBD treatments compared with the control (Figure 8C,D, F(3, 8) = 128.122, 100 ± 10.65% vs. 264.34 ± 28.57%, *p* < 0.001; 100 ± 10.65% vs. 191.28 ± 25.39%, *p* = 0.002). Moreover, the Tom 20 levels were significantly increased after cotreatment with CBD and Aβ compared with those after treatment with Aβ alone (Figure 8C,D, F(3, 8) = 128.122, 264.34 ± 28.57% vs. 304 ± 29.29%, *p* = 0.031). ROS production is related to mitochondrial toxicity. It was observed that CBD treatment decreased ROS generation (DCF fluorescence) and MMP (TMRE fluorescence) in the cells treated with Aβ compared with those treated with Aβ alone. Rapamycin further reduced the fluorescence of DCF (Figure 8E,F, DCF fluorescence: F(4, 56) = 40.835, Aβ vs. Aβ + CBD: 1.29 ± 0.23% vs. 0.81 ± 0.11%, *p* < 0.001; Aβ vs. Aβ + CBD + rap: 1.29 ± 0.23% vs. 0.43 ± 0.08%, *p* < 0.001; Aβ + CBD vs. Aβ + CBD + rap: 0.81 ± 0.11% vs. 0.43 ± 0.08%, *p* = 0.001; TMRE fluorescence: F(4, 55) = 4.503, Aβ vs. Aβ + CBD: 0.97 ± 0.07% vs. 1.11 ± 0.1%, *p* = 0.029; Aβ vs. Aβ + CBD + rap: 0.97 ± 0.07% vs. 1.19 ± 0.16%, *p* = 0.012). 

The next step was to determine how CBD affected the microglial metabolism under pathological conditions. LPS stimulation was similar to microglia activation in AD [33]. The LPS-induced inhibition of ATP production was significantly rescued upon CBD treatment (Figure S6C). Together, these results demonstrated that CBD enhances energy metabolism by reducing ROS production and MMP, improving mitochondrial function, and increasing ATP levels in microglial cells.

## 3. Discussion

The present study explored the contribution of TRPV2 and its agonist CBD on microglial Aβ phagocytosis and clearance. It was revealed that TRPV2 levels were decreased in AD patients and APP/PS1 mice. Moreover, CBD was observed to enhance microglial Aβ phagocytosis and clearance via the TRPV2 channel. Consistent with this, the expression levels of phagocytosis-related receptors in microglia were observed to be increased. Moreover, CBD could induce autophagy by promoting TRPV2 activation and upregulating Akt. In addition, CBD improved energy metabolism via mitochondrial functions in the microglia cells. These results demonstrated that CBD enhances the ability of TRPV2-mediated Aβ phagocytosis and clearance in microglia via autophagy and energy metabolism.

The TRPV2 channel is expressed in the CNS and implicated in developing axon outgrowth via the mitogen-activated protein kinase (MAPK) signaling pathway [17]. The present study revealed that the expression of the TRPV2 protein increased progressively with aging. A previous study demonstrated through bioinformatics analysis that TRPV2 mediates the MCI pathological changes in the peripheral blood of patients [14]. In the present study also, the levels of the TRPV2 protein were significantly reduced in the cortex and hippocampus of the APP/PS1 AD mice brain. However, in the dataset of AD patients, TRPV2 was significantly reduced in the entorhinal cortex and hippocampal samples. Therefore, the results of the present study strongly suggest the role of TRPV2 in the progression of AD. Microglia are essential to the pathophysiology of AD via direct phagocytosis of Aβ and secretion of inflammatory cytokines [34]. TRPV2 expression could be detected in the microglia in the human dataset. In addition, TRPV2 protein expression levels were higher in microglia compared with neurons. Together, these results indicated that microglial TRPV2 could have an important role in the development of AD.

Most drugs that reduce the Aβ levels in AD are reported to fail due to side effects, such as verubecestat (BACE-1 inhibitor) and avagacestat (gamma-secretase inhibitor), warranting the development of safer drugs [8,9]. CBD is a natural component exhibiting neuroprotective, anti-inflammatory, and antioxidant effects, which render it suitable as a candidate drug for AD [20,21,22,23,24]. As a candidate drug for AD, CBD acts through the cannabinoid receptors CB1 and CB2 [20,21]. CBD also acts as a high-affinity agonist of TRPV2 through a hydrophobic pocket located between S5 and S6 helices of adjacent subunits [22]. TRPV2 is critical for phagocytosis in microglia cells via PKG/PI3K- and iNOS/NO-dependent signaling pathways [35]. In the present study, CBD treatment significantly increased the efficiency of FITC-Aβ42 uptake in microglial cells via the TRPV2 channel. Moreover, intracellular Aβ levels were increased after treatment with CBD. This microglial phagocytic capacity was the highest via TRPV2 activation, while it was inhibited upon TRPV2 knockdown or using a TRPV2 antagonist. CBD could penetrate the blood–brain barrier in the human brain endothelial cells via TRPV2 activation [36]. Together, these findings suggest that CBD treatment markedly improves microglial phagocytic capacity via the TRPV2 channel and promotes Aβ clearance in microglia cells, further corroborating the role of CBD as a candidate drug for AD.

It is well recognized that unwanted cellular debris is removed through phagocytosis under the control of phagocytic receptors [37,38]. These receptors are, therefore, critical for the phagocytosis of aggregated proteins upon upregulation, which delays neuropathology and reduces neurodegeneration [39]. In the present study, CBD significantly improved the mRNA expression of TRPV2 and Trem2, and also enhanced the levels of other microglial phagocytic receptors upon treatment with Aβ. In addition, TRPV2 or Trem2 knockdown could eventually rescue these phagocytic receptors from the effects of CBD treatment along with Aβ in microglia cells, although this phenomenon presented different tendencies. In particular, inflammatory cytokines play critical roles in the occurrence and development of AD [40,41]. Consistent with this, cotreatment with CBD and Aβ in the present study markedly reduced the mRNA expressions of Iba1 and IL-1β, which could also be inhibited through TRPV2 or Trem2 knockdown. These results suggested that microglial phagocytic receptors are indispensable for the TRPV2-mediated enhancement of Aβ phagocytosis and clearance by CBD and also for reducing the inflammatory cytokines. These different receptors and inflammatory factors might exhibit temporal or synergistic interplays, which have to be investigated in future studies. 

TRPV2 is controlled by IGF-1 via the PI3K-dependent signaling pathway [18,19]. The PDK1 or Akt inhibitor inhibited the CBD-caused enhancement of Aβ phagocytosis and also blocked the TRPV2 protein expression in microglia, while IGF-1 increased the TRPV2 expression in N2a cells, suggesting that TRPV2 was transported from the cytoplasm into the plasma membrane and improved Ca^2+^-dependent exocytosis via the PI3K pathway, thereby further upregulating microglial phagocytosis [35]. Microglia cells could ingest and clean neuron-released Aβ through selective autophagy [27]. The CBD-induced TRPV2-dependent autophagic process stimulated glioma stem-like cells in a PI3K/Akt signaling-dependent manner [28]. Therefore, CBD reduces the pathological process of AD by enhancing autophagy [29]. In the present study, CBD induced the protein expression of autophagic flux in microglia cells by promoting TRPV2 activation and Akt phosphorylation after treatment with Aβ in a time-dependent manner. This protein expression could be inhibited by the inhibitor of TRPV2. Furthermore, the fluorescence of p62, a receptor in autophagy, was markedly increased upon cotreatment with Aβ and CBD. Surprisingly, the reduction in ROS production and MMP induced by CBD and Aβ was aggravated upon treatment with rapamycin (autophagy activator). These results demonstrated that the upregulation of TRPV2 by CBD led to the induction of autophagy and clearance of Aβ in microglia cells.

Microglial phagocytosis requires huge amounts of energy as cytoskeleton reorganization is required [42]. ATP is also critical for microglial phagocytosis and Aβ clearance [32]. In the present study, ATP production was observed to be significantly increased in microglia cells under Aβ treatment and elevated further upon combination treatment with CBD, while the knockdown of TRPV2 markedly reduced the ATP levels induced by CBD and Aβ cotreatment. The ATP levels in the N2a cells were not altered upon these treatments. Tom is the important channel for nuclear-encoded mitochondrial proteins, and Tom 20 is one of the receptor subunits in mammalian cells, which is also a key parameter of mitochondrial function and could be used as an indicator of cell health [13,43,44]. Tom 20 protein expression was observed to be significantly increased, although both ROS production and MMP were reduced in CBD and Aβ co-treated microglia, indicating that CBD might act directly on mitochondria. In addition, the stimulation of pro-inflammatory LPS, which is similar to activated microglia in AD, could increase microglial ATP production [33]. Therefore, ATP is indispensable for TRPV2-mediated microglial Aβ phagocytosis and clearance via CBD. 

In summary, the present study revealed that TRPV2 protein expression was reduced in the cortex and hippocampus of the brain of APP/PS1 mice and AD patients. Moreover, CBD enhanced microglial Aβ phagocytosis via TRPV2 in a time-dependent manner, and this process increased the mRNA expression levels of phagocytosis-related receptors, while TRPV2 or Trem2 knockdown rescued this expression. TRPV2 was observed to mediate the microglial Aβ phagocytosis induced by CBD via the PDK1/Akt-dependent pathway, and autophagy is indispensable for TRPV2-mediated Aβ phagocytosis and clearance. Therefore, CBD treatment could attenuate neuroinflammation and improve mitochondrial function and ATP levels. Overall, the results of the present study demonstrated that TRPV2 is a potential therapeutic target in AD, while CBD is a promising drug candidate for AD.

## 4. Materials and Methods

### 4.1. Mice

Different ages of C57BL/6J female mice were provided by the Animal Center of Lanzhou University, whereas the APP/PS1 female mice overexpressing APP (K670N:M671L) together with PS1 (M146L) under 6 months were purchased from Tengxin Biotechnology (Chongqing, China). Mice were housed in pathogen-free facilities (5–6/cage) with unlimited access to standard rodent chow and clean water. The animals needed to adapt to the laboratory for 7 days prior to the study. All of the experimental procedures were approved by following recommendations on the care and use of experimental animals stated in the Northwest Normal University Ethical Committee for Human and Animal Experimentation Guidelines. The study complies with the ARRIVE guidelines developed by the NC3Rs [45].

### 4.2. Reagents and Antibodies

CBD was obtained from Refines Biotechnology (Chengdu, China). Protease inhibitor cocktail (# HY-K0010), Akt kinase inhibitor (# HY-10249A), rapamycin (# HY-10219), tranilast (# HY-B0195) and 2-aminoethoxydiphenyl borate (2APB) (# HY-W009724), capsaicin (# HY-10448), GSK2334470 (# HY-14981), and GSK2656157 (# HY-13820) were purchased from MCE. Dulbecco’s modified Eagle’s medium (DMEM), minimum essential medium (MEM), streptomycin, penicillin, Opti-MEM, and fetal bovine serum (FBS) were obtained from HyClone (Logan, UT, USA). Neurobasal medium and B27 supplement were obtained from Invitrogen. Human recombinant IGF-1 and DAPI were obtained from the Solarbio Co. of Beijing, China, and Lipofectamine 3000 was obtained from Invitrogen (Waltham, MA, USA). The TRPV2 (1:1000, polyclonal antibody, # ALO-ACC-032-50) was purchased from Alomone Labs (Jerusalem, Israel). The following antibodies were purchased from the Cell Signaling Technology Co. of Danvers, MA, USA: Akt (1:1000, polyclonal antibody, # 9272); phospho-Akt Ser473 (1:1000, polyclonal antibody, # 9271); LC3B (1:1000, polyclonal antibody, # 12741). Iba1 (1:200, polyclonal antibody, # 10904-1-AP), P62 (1:1000, polyclonal antibody, # 18420-1-AP), Beclin 1 (1:1000, polyclonal antibody, # 11306-1-AP), and Tom 20 (1:4000, polyclonal antibody, # 11802-1-AP) were obtained from Proteintech (Wuhan, China). GAPDH (1:1000, polyclonal antibody, # K106390M) was purchased from Solarbio (Beijing, China). The Aβ (6E10) monoclonal antibody was obtained from Bioss (Beijing, China). The host species of all antibodies were rabbit, and the target specificity was mouse. All other reagents and chemicals were purchased from Solarbio (Beijing, China).

### 4.3. Primary Cultures of Microglial Cells and Neurons

Primary microglia and neuron cultures were prepared as described in previous reports [35,46]. Embryonic day 15,5 (E15,5) embryos from cortical tissues were obtained from the primary neuron cultures, while 1-day-old pups were obtained from primary microglia cultures. The obtained neurons were seeded at a density of 2 × 10^5^ cells/cm^2^ into the well of 6-well plates that were coated with 50 µg/mL poly-D-lysine or at a density of 5 × 10^4^ cells/cm^2^ into the wells of 24-well plates and then cultured in neurobasal medium containing 2% B27 for 6 days under normoxia conditions. The primary microglial cells were cultured in T-25 flasks containing DMEM complete medium under normal conditions of 5% CO_2_. The entire spent medium was replaced every 3 days, and confluency was achieved after nearly 12 days of in vitro culture. The cell cultures were incubated inside a shaker incubator at 200 rpm for 18 h, followed by collecting the supernatants, which were subjected to centrifugation at 900 g for 4 min. The obtained pellets containing isolated microglia were resuspended in DMEM complete media and plated at a density of 5 × 10^4^ cells/cm^2^ in different plates for subsequent experiments.

### 4.4. Cell Culture

Mouse neuroblastoma N2a cells procured from Procell (CL-0168) were cultured in the MEM medium. The microglia BV-2 cell line, provided kindly by Prof. Shengxiang Zhang from Lanzhou University, was cultured in the high-glucose DMEM medium. The media for the culture of both kinds of cells were supplemented with 10% fetal bovine serum and 1% penicillin (70 mg/L) and streptomycin (100 mg/L). The cells were cultured at 37 °C inside an incubator (Yiheng Technology, Shanghai, China) with a 5% CO_2_ atmosphere and 95% humidity. The Akt inhibitor, rapamycin, tranilast, 2-APB, capsaicin, GSK2334470, and GSK2656157, each dissolved in DMSO, were used for treating samples, with the final concentration of DMSO remaining less than 0.1%. CBD was first dissolved in methanol and then diluted with serum-free medium, while IGF-1 was diluted in DMEM for use in experiments.

### 4.5. Western Blot

Dissected brain tissues were homogenized in a 10-fold excess volume of ice-cold RIPA protein lysis buffer (Solarbio, Beijing, China) containing 1% protease inhibitor cocktail via the Polytron homogenizer. After various treatments, cells were scrapped from wells and lysed with the same ice-cold lysis buffer. The lysates were left on ice and centrifuged at 4 °C for 15 min at 14,000× *g*, and the supernatants were aliquoted and preserved at −20 °C. The protein concentrations were measured by the Bradford method using a commercially available kit (# PC0010, Solarbio, Beijing, China). Proteins (40 μg) were subjected to 7.5–15% SDS-PAGE in a gel electrophoresis system (Tanon, Shanghai, China) and transferred onto nitrocellulose membranes (Whatman, Maidstone, UK) using the wet transfer system. The membranes were then blocked in 10% nonfat skimmed milk prepared by 0.2% Tris-buffered saline/Tween 20 (TBST) for 1 h at room temperature, and then probed with primary antibodies in 1% BSA/1 × TBST according to the manufacturer’s instructions. After being washed with TBST, the membranes were further immunoblotted with the corresponding HRP-conjugated secondary antibodies (# bs-0295G-HRP, Bioss, Beijing, China) prepared with 5% milk for 1 h at room temperature. The membranes were treated with Millipore Immobilon Western Chemiluminescent HRP substrate (# WBKLS0050), either exposed to Super RX Fujifilm or detected using a ChemiDoc MP Imaging System (VILBER, SN:18200692, Feldkirchen, Germany), and quantified using the ImageJ software. The band densitometry values of TRPV2 were normalized with GAPDH (inner control) or total protein levels of immunoreactivity in the same lane to correct for any loading or transfer differences among samples.

### 4.6. Preparation of Amyloid-Beta

Preparation of amyloid-beta was performed as described in a previous study [47]. An amount of 1 mg of amyloid peptides (1-42) was synthesized from Shanghai Gil Biochemical Co. (# 052487, Shanghai, China). The peptides were resuspended in 20 μL of DMSO, vortexed, and incubated for 5 min in shaking and further added with 424 μL of PBS until a final concentration is 500 μmol. The solution was maintained for 24 h at 37 °C and centrifuged for 10 min at 14,000× *g* at 4 °C, and the supernatant including soluble Aβ1-42 oligomers was collected and biochemically assessed using bicine/Tris/urea SDS-PAGE by Western blot. 

### 4.7. Immunofluorescence

Microglia cells were cultured on poly-D-lysine coverglass for the indicated number of experimental days in 24-well plates and then fixed with 4% paraformaldehyde (Biosharp # BL539A, Beijing,, China) in phosphate-buffered saline (PBS) for 20 min at room temperature, fixative solution was washed three times with PBS for 10 min each. The cells were permeabilized with 0.01% Triton X-100 (Solarbio # T8200) in PBS for 7 min and blocked using 5% normal goat serum containing 0.01% Triton X-100 in PBS for 1 h. P62 and IBA1 antibodies were diluted at 1:200 in PBS supplemented with 0.01% Triton X-100 and 1% normal goat serum and incubated overnight at 4 °C. Cells were followed by three washes with PBS for 10 min each. Conjugated to Alexa Fluor 594 (Proteintech # SA000 13, Wuhan, China), fluorescent goat anti-rabbit secondary antibody was used at a concentration of 1:400 for 90 min. Microglia were washed three times with PBS, and nuclei were stained with DAPI for 30 min at room temperature. Coverslips were washed again and then mounted onto microscope slides with Fluoromount-G (Solarbio # S2100) for further analysis. Immunofluorescence sections were taken using a fluorescence microscope (Leica, # DM6B/DFC700T) under × 20 and × 40 magnification. The images were processed and analyzed with ImageJ 1.42q (Wayne Rasband, National Institutes of Health) and Fiji (http://pacific.mpicbg.de/wiki/index.php/Main_Page, 29 April 2019) software. For the quantitative analyses, the immunofluorescence density was analyzed by a blinded experimenter to determine the positive expression in at least five selected images per treatment, and the DAPI stain (1 μg/mL) determined the nuclear area for normalization with control.

### 4.8. RNA Extraction and Quantitative PCR

The total RNA was extracted from the microglia cells using TRIzol (Solarbio, MF034-01) according to the manufacturer’s instructions. In brief, 0.5 mL of TRIzol was added to each well of a 6-well plate containing the cells washed with PBS. The plate was incubated at room temperature for 5 min, and then 100 µL of ice-cold chloroform (Solarbio # P1025) was added to the well, followed by vigorously shaking for 30 s and leaving the plate undisturbed for 3 min. The homogenates were centrifuged at 13,400× *g* for 15 min at 4 °C. The upper aqueous phase was carefully transferred to a tube containing 0.5 mL of acidic phenol–chloroform, followed by shaking the tube for 30 s and then incubating it for 5 min. The samples were centrifuged at 13,400× *g* for 15 min at 4 °C. The upper aqueous phase was again transferred to an empty tube containing 0.5 mL of isopropanol, followed by 30 s of shaking and then 10 min of incubation. After centrifugation at the same conditions, the supernatants were collected and mixed with 75% RNase-free ethanol. The samples were centrifuged again, and the obtained supernatants were mixed with 75% RNase-free ethanol. The sample pellets were then air-dried at room temperature and resuspended in 50 µL of diethylpyrocarbonate (DEPC) water (Solarbio # R1600). The purified RNAs were quantified, and their quality was assessed using a NanoDrop 2000 UV–VIS spectrophotometer (Thermo Fisher Scientific, Wilmington, DE, USA). Subsequently, the cDNA was obtained from the RNA samples using a RevertAid First Strand cDNA Synthesis Kit (Thermo Scientific # K1622). An amount of 1 µg of RNA was reverse-transcribed at a reaction volume of 20 µL using oligo(dT)s according to the manufacturer’s instructions.

The synthesized cDNA (10 ng) was amplified using specific primers in 10 µL of quantitative real-time PCR (RT-qPCR) reaction using a SYBR green PCR master mix (Tiangen # FP205). The primer pairs used were synthesized based on previous reports and are presented in Table 1. The gene expression was detected in a real-time detection system (Thermo Scientific), which evaluated 96 wells containing gene samples along with other positive and negative controls. The reaction conditions comprised an initial denaturation at 95 °C for 10 min, one reaction cycle at 95 °C for 15 s, another cycle at 50–65 °C (depending on the primer) for 1 min, and a final extension at 72 °C for 1 min. A total of 40 PCR cycles were performed in 20 µL of reaction volume. The mRNA expression of each gene was evaluated in duplicate and quantified relative to the expression of the reference gene GAPDH. The normalized expressions of the targeted genes were calculated using the 2^−ΔΔCt^ method [48].

### 4.9. Transfection of Cells with Trem2 and TRPV2 siRNA

The negative siRNA (sense UUCUCCGAACGUGUCACGUTT; antisense ACGUGACACGUUCGGAGAATT) and the TREM2 siRNA (sense UUCUCCUGAGCAAGUUUCUUG; antisense CAUCACUCUGAAGAACCUCCA) were synthesized and obtained from Sangon Biotech (Shanghai, China) [13]. The TRPV2 siRNA was obtained from Thermo Fisher Scientific (Stealth siRNA no. MSS-212426; Waltham, MA, USA). The RNA transfection experiments were conducted as follows: Briefly, the microglia cells were plated at a density of 2.0 × 10^5^ in the 6-well plates and at a density of 5 × 10^4^ in the 96-well plates. In the Trem2 siRNA and negative siRNA groups, 50 nmol of TRPV2 siRNA oligonucleotide or the negative control, along with the TRPV2 siRNA, was dissolved in DEPC water (20 nmol), while 8 µL of siRNA was diluted in 592 µL of Opti-MEM. Lipofectamine 3000 (20 µL) was diluted in 580 µL of Opti-MEM, preincubated at room temperature for 5 min, and then mixed and incubated for an additional 20 min. The control was prepared by dissolving siRNA in DEPC water in Opti-MEM; the mixture contained only 20 µL of Lipofectamine 3000 in 580 µL of Opti-MEM. After the cell culture media were replaced with Opti-MEM, the entire mixture was added to the wells (150 μL for 6 wells and 15 μL for 96 wells). The cells were incubated for 24 h, followed by replacing half of the medium with fresh DMEM complete medium and then culturing again for 48 h. The mRNA levels in the TRPV2 and Trem2 group samples were analyzed using qRT-PCR.

### 4.10. Phagocytosis Assays

The phagocytosis of aggregated Aβ1–42 was analyzed as described in a previous study [13]. Briefly, FITC-Aβ1–42 (Gil Biochemical, # 250857, Shanghai, China) was allowed to aggregate for 24 h at 37 °C. The microglia cells were plated at a density of 2 × 10^4^ cells per well in the poly-D-lysine-coated wells of 96-well plates, followed by an overnight culture. After CBD (10 μmol) treatment for 24 h, Aβ was added to a final concentration of 1 µg/mL, followed by culture for an indicated time at normal conditions. Prior to measurements, the culture medium was removed, and the cells were washed twice with a fresh DMEM complete medium to remove extracellular Aβ. Finally, the plates were protected from light, and fluorescence was measured in a multifunctional microplate reader (Spark 10M, Tecan, Zurich, Switzerland) at a 485 nm excitation/538 nm emission wavelength.

In addition, Aβ phagocytosis was verified using immunocytochemistry and Western blotting. Briefly, the microglia cells were plated at a density of 1 × 10^5^ cells in the poly-D-lysine-coated wells of 24-well plates. After Aβ and CBD treatments, the cells were subjected to immunofluorescence and Western blot analyses. Anti-Iba1 antibody was used as a marker for cell shape, while DAPI (1 μg/mL) was used for staining the nuclei. Cell images were acquired and analyzed using the ImageJ software. The average intensity was presented as an intracellular FITC-Aβ1–42 signal and normalized to the vehicle group.

In the Western blot analysis, the cells were lysed using the 1 × loading buffer (25 mmol Tris-HCl pH 6.8, 10% glycerol, 2% SDS, 0.0025% bromophenol blue, 1% β-mercaptothanol). The supernatants from the lysed cell mixture were collected and mixed with equal volumes of methanol. The supernatant/methanol mixture was then added to trichloromethane (1:4 volume) and agitated thoroughly by upside-down mixing. The mixtures were then centrifuged at 14,000 × *g* for 15 min at 4 °C, which generated three layers in the centrifuged mixture. The middle layer from these three layers was collected and air-dried. Intracellular Aβ and supernatant Aβ were lysed using 1 × loading buffer and then evaluated using Western blotting.

### 4.11. MTT Reduction Assay

Cell viability was determined by performing the MTT reduction assay. Briefly, the 3-[4,5-dimethylthiazol-2-yl]2,5-diphenyltetrazolium bromide salt (MTT, Solarbio #M1020) was added to the wells of a 96-well plate containing 10 µL of cell culture and 90 µL of complete medium, followed by 4 h of incubation. The culture medium was aspirated completely, and the resulting formazan crystals were dissolved by adding 100 µL of DMSO. The absorbance intensity was measured at 490 nm inside a multifunctional microplate reader (Spark 10M, Tecan, Zurich, Switzerland).

### 4.12. Mitochondrial Membrane Potential (MMP)

The mitochondrial membrane potential of the microglia cells was determined using the TMRE dye (MCE # HY-D0985A). Briefly, the cells were differentiated through 4 days of treatment with retinoic acid (10 µmol; Sigma-Aldrich, R2625) containing 1% fetal bovine serum, followed by pretreatment with Aβ for 24 h and then treatment with CBD for 24 h. Subsequently, 300 nmol of TMRE was added 30 min prior to the termination of the experiment. Finally, the cells were washed with PBS, and changes in the mitochondrial membrane potential (ΔΨm) were measured based on the red fluorescence of TMRE inside a multifunctional microplate reader (Spark 10M, Tecan, Zurich, Switzerland).

### 4.13. Reactive Oxygen Species (ROS)

ROS production was monitored inside a multifunctional microplate reader using 2’, 7′-dichlorodihydrofluorescein diacetate (DCFH2-DA) (Sigma # 4091-99-0m, St. Louis, MO, USA). The differentiated microglia cells were pretreated with Aβ for 24 h and then stimulated with 250 nmol of rapamycin for 1 h. This was followed by treatment with CBD for 24 h. DCFH2-DA (10 µmol) was added 30 min prior to the termination of the experiment. The cells were subsequently washed with PBS, and fluorescence was measured inside the multifunctional microplate reader (Spark 10M, Tecan, Zurich, Switzerland) at 488 nm excitation/525 nm emission wavelengths.

### 4.14. Mitochondrial Extraction and Measurement of ATP

The ATP activities and mitochondrial extraction of different cells were tested using the appropriate kits (ATP: # A095, Jiancheng Bioengineering Institute, Nanjing; mitochondrial extraction kit: SM0020, Solarbio, China) in accordance with the guidelines provided by the manufacturer. 

### 4.15. Statistical Analysis

Data were presented as the means ± standard error and analyzed using GraphPad software (GraphPad Software Inc., La Jolla, CA, USA) and SPSS software (SPSS Inc., Chicago, IL, USA). Unpaired two-tailed Student’s *t*-test analysis determined the statistical significance of the difference between two groups. One-way ANOVA followed by post hoc Tukey’s test method was applied to calculate the difference of multiple groups. Data points that fell beyond two standard deviations from the mean were considered statistical outliers and excluded from further analysis. The *p*-values of <0.05, <0.01, and <0.001 were considered significant and were indicated in the figures.

## Figures and Tables

**Table 1:** Primers and their sequences for fluorescence quantitative PCR.

Primer	Sequence
TRPV2 Forward	GGTATGGGTGAGCTGGCTTTT
TRPV2 Reverse	AGGACGTAGGTGAGGAGGAC
TREM2 Forward	CTGATCACAGCCCTGTCCCAA
TREM2 Reverse	CGTCTCCCCCAGTGCTTCAA
Gpr34 Forward	CTTCAGGAAAGCTTCAACTC
Gpr34 Reverse	GTAACTATCAGGAGGAGAGC
P2Y6 Forward	GTGAGGATTTCAAGCGACTGC
P2Y6 Reverse	TCCCCTCTGGCGTAGTTATAGA
IL-4 Forward	GGTCTCAACCCCCAGCTAGT
IL-4 Reverse	GCCGATGATCTCTCTCAAGTGAT
IL-10 Forward	GCTCTTACTGACTGGCATGAG
IL-10 Reverse	CGCAGCTCTAGGAGCATGTG
Iba1 Forward	ATCAACAAGCAATTCCTCGATGA
Iba1 Reverse	CAGCATTCGCTTCAAGGACATA
Cr3 Forward	AATTGAGGGCACGCAGACA
Cr3 Reverse	GCCCAGCAAGGGACCATTAG
TNFα Forward	ACTCCAGGCGGTGCCTATGT
TNFα Reverse	GTGAGGGTCTGGGCCATAGAA
IL-1β Forward	TCCAGGATGAGGACATGAGCAC
IL-1β Reverse	GAACGTCACACACCAGCAGGTTA
IL-6 Forward	CCACTTCACAAGTCGGAGGCTTA
IL-6 Reverse	CCAGTTTGGTAGCATCCATCATTTC
GAPDH Forward	TGTGTCCGTCGTGGATCTGA
GAPDH Reverse	TTGCTGTTGAAGTCGCAGGAG

## Data Availability

Not applicable.

## Supplementary Materials

The following supporting information can be downloaded at: https://www.mdpi.com/article/10.3390/ijms23105367/s1.
