# Supplementary material for: Cannabidiol Enhances Microglial Beta-Amyloid Peptide Phagocytosis and Clearance via Vanilloid Family Type 2 Channel Activation
Source: Int J Mol Sci. 2022 May 11;23(10):5367. doi: 10.3390/ijms23105367 (PMC9140666; doi:10.3390/ijms23105367)
Supplement: Supplementary file 1 [file ijms-23-05367-s001.zip › ijms-1694191-supplementary.pdf]

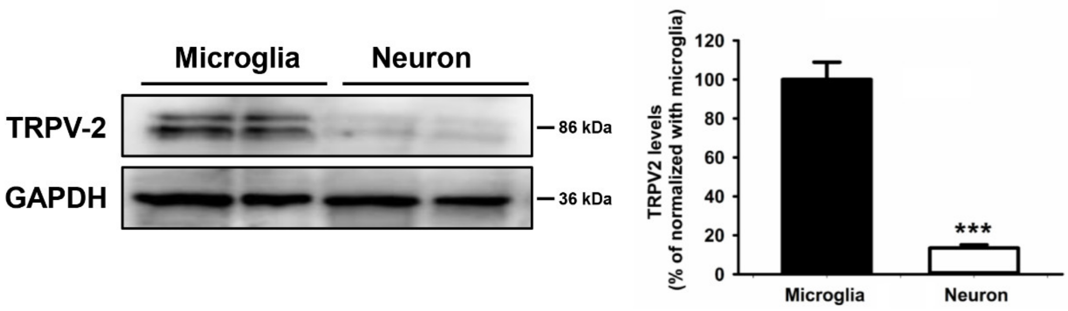

Figure S1. TRPV2 expression levels were different in neuron and microglia. (B) The protein expression levels of TRPV2 were measured on neuronal and microglial extracts. The data are represented as the mean ± SEM (n = 3/group). *** $p < 0.001$ is compared to the neuron by the Student's t-test.

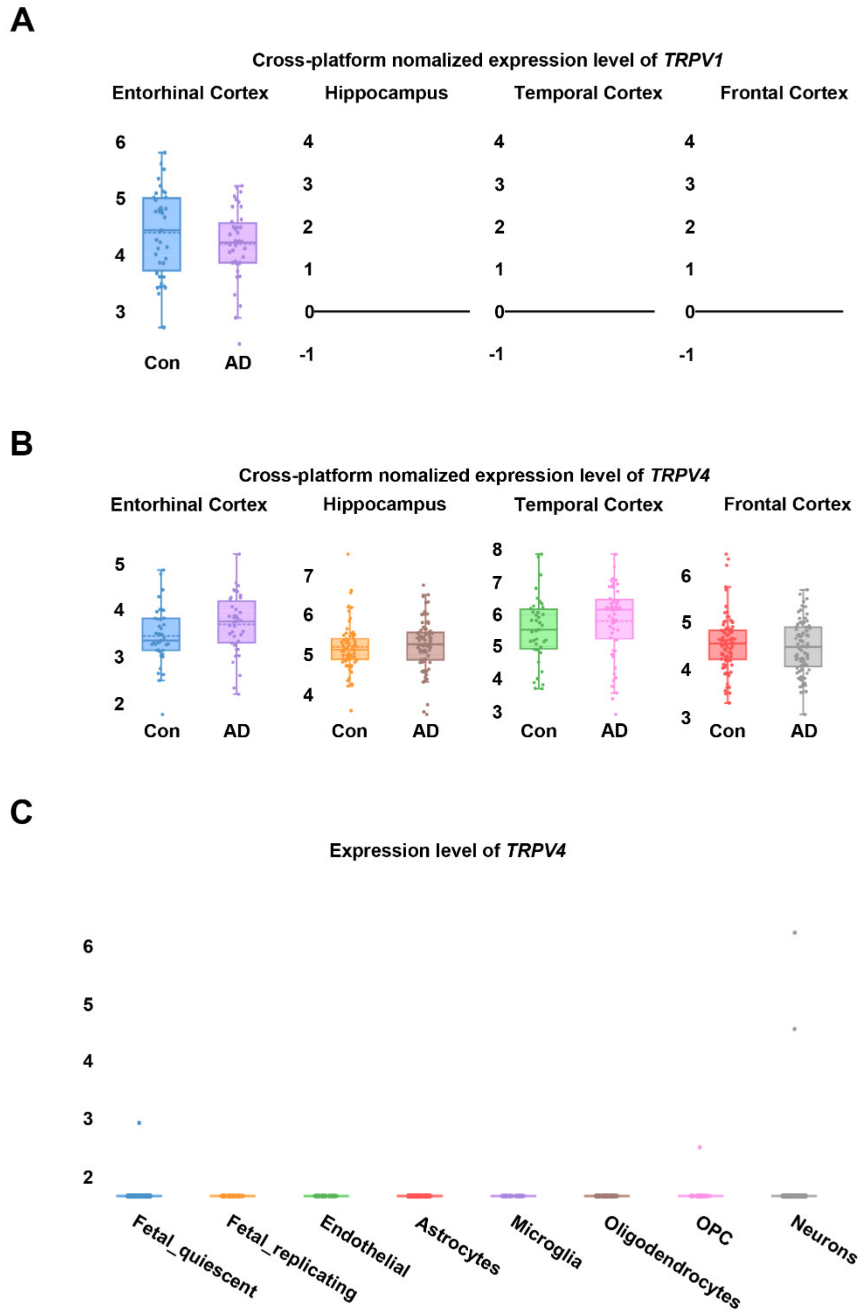

Figure S2. TRPV1 and TRPV4 expression levels in AD patients. (A) Transcriptional expression level of TRPV1 in entorhinal, temporal and frontal cortex and hippocampus tissues of patients with AD in the cross-platform database. (B) Transcriptional expression level of TRPV4 in entorhinal, temporal and frontal cortex and hippocampus tissues of patients with AD in the cross-platform database. (C) Transcriptional expression level of TRPV4 in different cells (endothelial, microglia, oligodendrocytes and neurons) were different in the GSE67835 database.

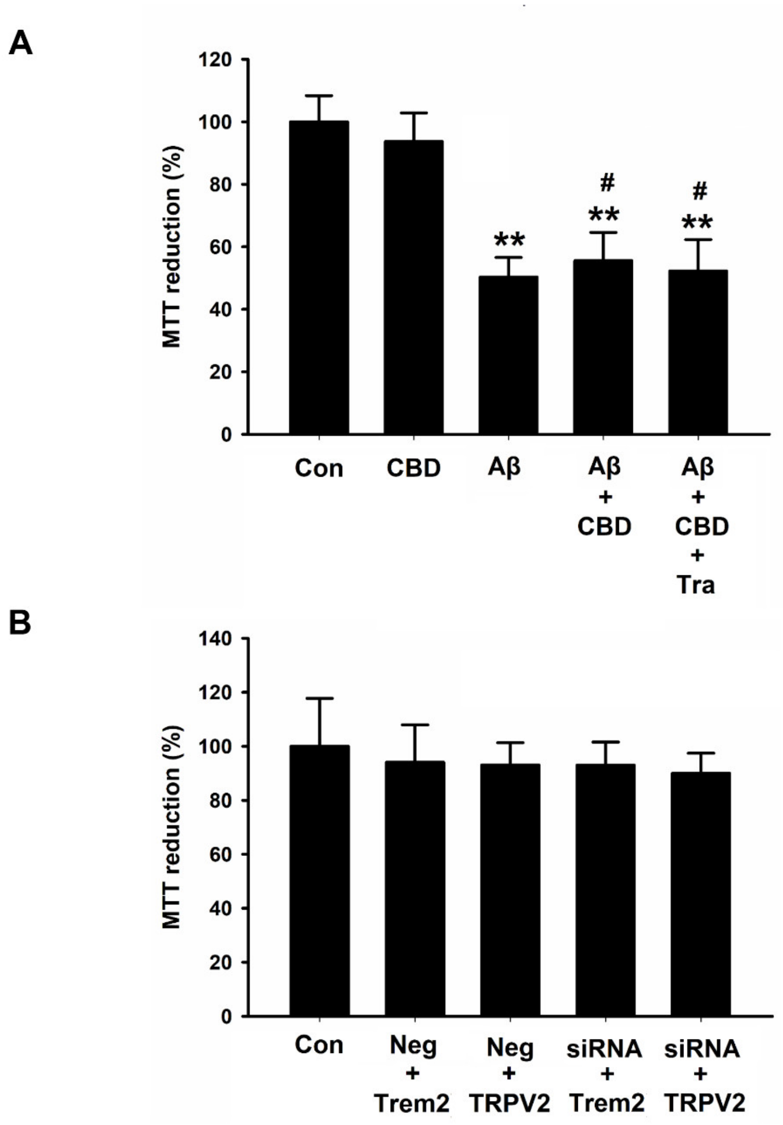

Figure S3. Microglial survival was different feature after siRNA and inhibitor. (A) BV2 microglia were pre-added with tra (tranilast 75 μM), and treated with or without CBD after 24 hours (n = 12), (B) BV2 microglia were treated with negative siRNA, TRPV2 and Trem2 siRNA (n = 6), the cell viability was determined with the MTT reduction assay and was expressed as a percentage of the untreated cells. ** $p < 0.01$ are compared to the control by the Tukey's test; # $p < 0.05$ is compared to Aβ group by the Tukey's test.

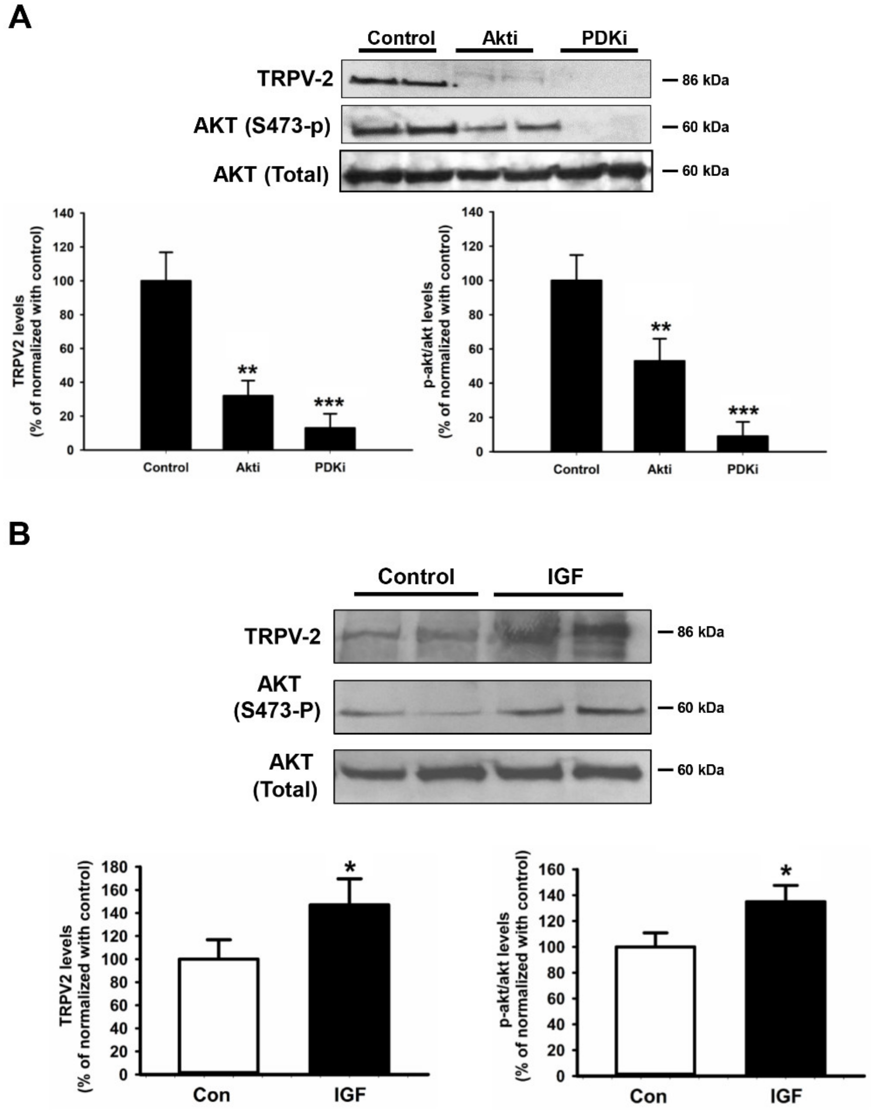

Figure S4. TRPV2 protein expression was changed following PI3K/Akt signaling pathway. (A) BV2 microglia were treated with akti (Akt inhibitor 10 μM), PDK1i (PDK1 inhibitor: GSK2334470 1μM) after 24 hours. (B) N2a cells were treated with IGF-1 (50 ng/ml) after 4 hours, the TRPV2 protein levels and the phosphorylation of Akt were determined by western blot. Band densitometry quantification of TRPV2 and the phosphorylation of Akt were normalized to total Akt levels. The data are represented as the mean ± SEM (n=4). * $p < 0.05$, ** $p < 0.01$ and *** $p < 0.001$ are compared to control by the Tukey's test.

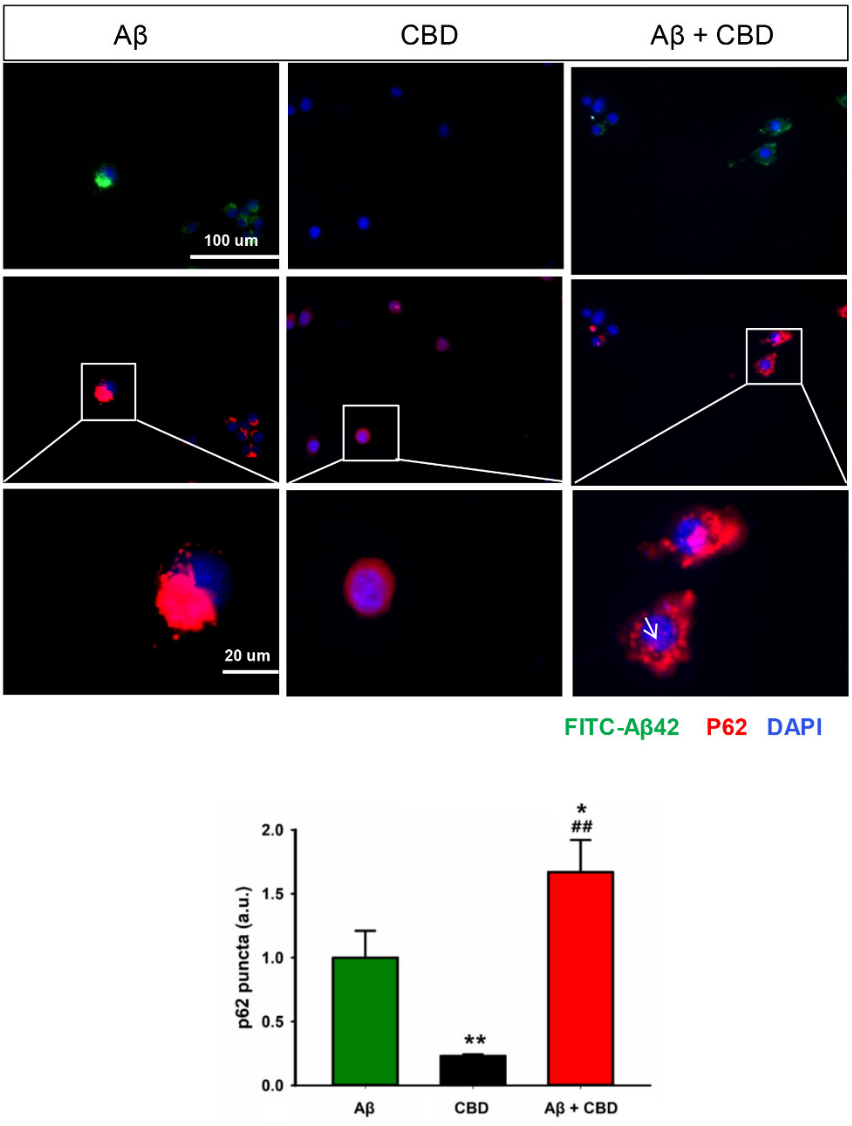

Figure S5. CBD induced p62 expression in microglial cells. After the primary microglia were treated with Aβ for 24 hours, representative immunofluorescence staining of the p62 in each group were observed in the sections (arrows in the images denote the p62). The intensity of the signals was quantified and expressed as a percentage of the controls using ImageJ software (n = 30 to 46 per group). a.u., arbitrary units. The data are represented as the mean ± SEM. * $p < 0.05$, and ** $p < 0.01$ are compared to the controls by the Tukey's test.

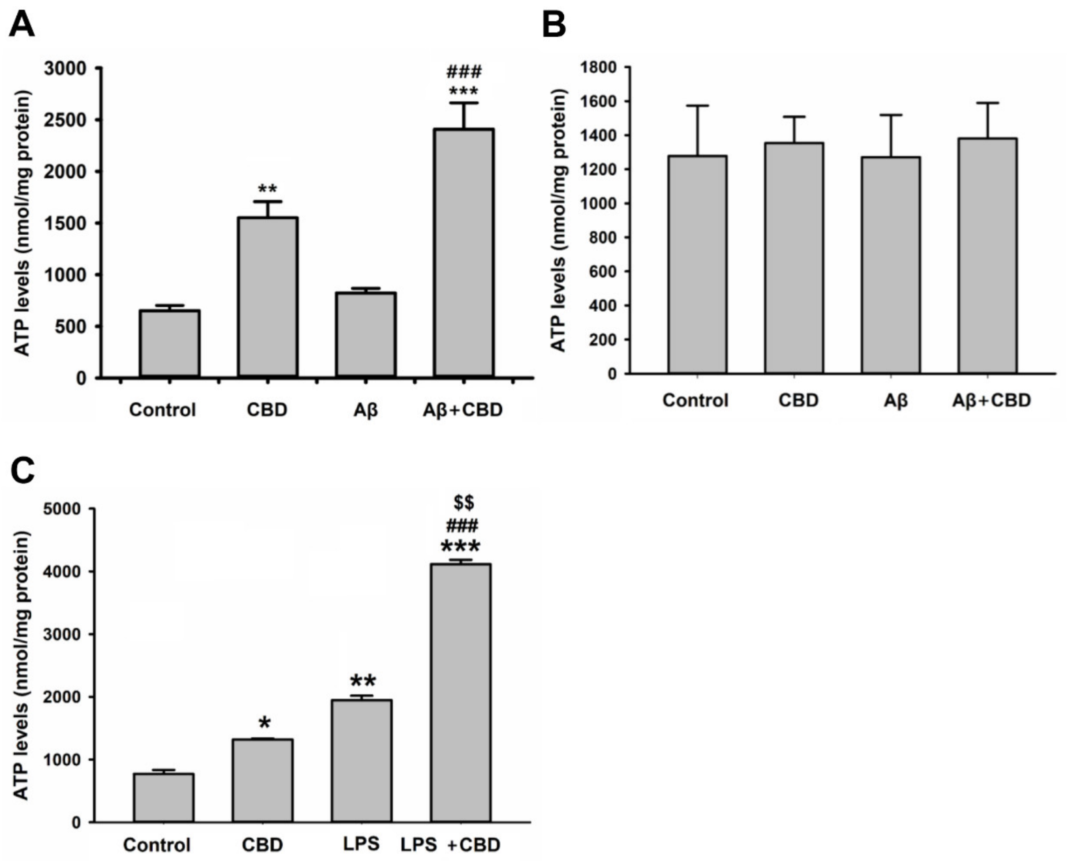

Figure S6. Total ATP production of BV2 and N2a cells under different conditions. (A) N2a cells were cultured at 1×10^5 on poly-D-lysine in 6-well plates with MEM medium, and after 24 hours, half of medium was removed. BV2 cells were plated at 1×10^5 with DMEM complete medium, and after 24 hours, the co-cultured cells were treated with 1 μg/ml FITC-Aβ42, CBD (10 μM) was added to medium for 24 hours. (B) Total ATP production of N2a cells under different conditions as indicated. Aβ and/or CBD were added to the cells for 24 hours before measurement. (C) Total ATP production of BV2 cells under different conditions as indicated. LPS (1 μg/ml) and/or CBD were added to the cells for 24 hours before measurement. The data are represented as the mean ± SEM (n=4). * $p < 0.05$, ** $p < 0.01$ and *** $p < 0.001$ are compared to the controls by the Tukey's test; ### $p < 0.001$ is between CBD and CBD + Aβ by the Student's t-test; $$ $p < 0.01$ is between Aβ and CBD + Aβ by the Student's t-test.

Table S1. Cannabidiol enhances microglial beta-amyloid peptides phagocytosis and clearance via vanilloid family type 2 channel activation.

Figure	Statistical analysis	Number of observations	T or F values	VS	P values
Fig. 1A	One-way ANOVA with Tukey's *post hoc* test	4	$F(5, 32) = 7.475$	E15 vs. E17<br>E15 vs. P1<br>E15 vs. P3<br>E15 vs. M4<br>E15 vs. M6	$P = 0.988$<br>$P = 0.206$<br>$P = 0.024$<br>$P = 0.012$<br>$P = 0.001$
Fig. 1B	Unpaired, two-tailed student's *t* test analysis	4	$t(6) = 2.916$<br>$t(6) = 4.432$	CX: WT vs. APP<br>HP: WT vs. APP	$P = 0.027$<br>$P = 0.004$
Fig. 1C	Date from AlzData web server (www.alzdata.org)	19		EC: Con vs. AD<br>HP: Con vs. AD<br>TC: Con vs. AD<br>FC: Con vs. AD	$P = 0.035$<br>$P = 0.181$<br>$P = 0.22$<br>$P = 0.067$
Fig. 1D	Date from AlzData web server (www.alzdata.org)	19		EC: Con vs. AD	$P = 0.055$
Fig. 1E	Date from AlzData web server (www.alzdata.org)	19		HP: Con vs. AD	$P = 0.017$
Fig. 2B	One-way ANOVA with Tukey's *post hoc* test	4	$F(2, 9) = 35.622$	Aβ vs. CBD<br>Aβ vs. CBD + Aβ<br>CBD vs. CBD + Aβ	$P = 0.004$<br>$P = 0.011$<br>$P < 0.001$
Fig. 2C	Unpaired, two-tailed student's *t* test analysis	3	$t(10) = -1.812$<br>$t(10) = -2.721$<br>$t(10) = -2.828$<br>$t(10) = -3.003$<br>$t(10) = -3.841$	1h: Aβ vs. Aβ + CBD<br>3h: Aβ vs. Aβ + CBD<br>6h: Aβ vs. Aβ + CBD<br>16h: Aβ vs. Aβ + CBD<br>24h: Aβ vs. Aβ + CBD	$P = 0.11$<br>$P = 0.022$<br>$P = 0.018$<br>$P = 0.013$<br>$P = 0.003$
Fig.	Unpaired,	4	$t(6) = 5.365$	Sup: Aβ vs. Aβ +	$P = 0.002$

2E	two-tailed student's *t* test analysis		t(6) = -2.87	CBD<br>Lys: Aβ vs. Aβ + CBD	*P* = 0.028
Fig. 3A	One-way ANOVA with Tukey's *post hoc* test	6	F(4, 25) = 23.792	Con vs. Aβ<br>Con vs. Aβ + CBD<br>Con vs. Aβ + 2-PAB<br>Con vs. Aβ + CAP<br>Aβ vs. Aβ + CBD<br>Aβ vs. Aβ + 2-PAB<br>Aβ vs. Aβ + CAP	*P* = 0.001<br>*P* < 0.001<br>*P* < 0.001<br>*P* = 0.003<br>*P* = 0.002<br>*P* = 0.013<br>*P* = 0.0045
Fig. 3B	One-way ANOVA with Tukey's *post hoc* test	6	F(3, 20) = 5.118	WT + Aβ vs. Aβ + CBD<br>siRNA + Aβ vs. siRNA + Aβ + CBD<br>WT + Aβ + CBD vs. siRNA + Aβ<br>WT + Aβ + CBD vs. siRNA + Aβ + CBD	*P* = 0.045<br>*P* = 0.981<br>*P* = 0.011<br>*P* = 0.026
Fig. 3C	One-way ANOVA with Tukey's *post hoc* test	6	F(3, 20) = 26.223	Con vs. Aβ<br>Con vs. Aβ + CBD<br>Con vs. Aβ + CBD + Tra<br>Aβ vs. Aβ + CBD<br>Aβ + CBD vs. Aβ + CBD + Tra	*P* = 0.002<br>*P* < 0.001<br>*P* = 0.003<br>*P* = 0.004<br>*P* = 0.001
Fig. 3D	One-way ANOVA with Tukey's *post hoc* test	6	F(3, 20) = 16.863	Con vs. Aβ<br>Con vs. Aβ + CBD<br>Con vs. Aβ + CBD + Akti<br>Aβ vs. Aβ + CBD<br>Aβ + CBD vs. Aβ + CBD + Akti	*P* = 0.002<br>*P* < 0.001<br>*P* = 0.004<br>*P* = 0.004<br>*P* = 0.01
Fig. 3E	One-way ANOVA with Tukey's *post hoc* test	6	F(3, 20) = 38.978	Con vs. Aβ<br>Con vs. Aβ + CBD<br>Con vs. Aβ + CBD + PDK1i<br>Aβ vs. Aβ + CBD<br>Aβ + CBD vs. Aβ + CBD + PDK1i	*P* = 0.002<br>*P* < 0.001<br>*P* = 0.001<br>*P* = 0.004<br>*P* < 0.001
Fig. 3F	One-way ANOVA with Tukey's	6	F(3, 20) = 25.018	Con vs. Aβ<br>Con vs. Aβ + CBD<br>Con vs. Aβ + CBD + PERKi	*P* = 0.002<br>*P* < 0.001<br>*P* = 0.003

	post hoc test			Aβ vs. Aβ + CBD<br>Aβ + CBD vs. Aβ + CBD + PERKi	P = 0.004<br><br>P = 0.002
Fig. 4 TRPV2	One-way ANOVA with Tukey's *post hoc* test	6	F(3, 20) = 12.335	Con vs. CBD<br>Con vs. Aβ<br>Con vs. Aβ + CBD<br>Aβ vs. Aβ + CBD	P = 0.013<br>P = 0.991<br>P = 0.002<br>P = 0.001
Fig. 4 Trem2	One-way ANOVA with Tukey's *post hoc* test	6	F(3, 20) = 67.094	Con vs. CBD<br>Con vs. Aβ<br>Con vs. Aβ + CBD<br>Aβ vs. Aβ + CBD	P = 0.008<br>P = 0.005<br>P < 0.001<br>P = 0.001
Fig. 4 Iba1	One-way ANOVA with Tukey's *post hoc* test	6	F(3, 20) = 56.861	Con vs. CBD<br>Con vs. Aβ<br>Con vs. Aβ + CBD<br>Aβ vs. Aβ + CBD	P = 1.000<br>P < 0.001<br>P < 0.001<br>P = 0.002
Fig. 4 IL-1β	One-way ANOVA with Tukey's *post hoc* test	6	F(3, 20) = 8.753	Con vs. CBD<br>Con vs. Aβ<br>Con vs. Aβ + CBD<br>Aβ vs. Aβ + CBD	P = 0.773<br>P = 0.001<br>P = 0.04<br>P = 0.004
Fig. 4 IL-6	One-way ANOVA with Tukey's *post hoc* test	6	F(3, 20) = 16.541	Con vs. CBD<br>Con vs. Aβ<br>Con vs. Aβ + CBD<br>Aβ vs. Aβ + CBD	P = 0.036<br>P = 0.321<br>P = 0.005<br>P = 0.012
Fig. 4 IL-4	One-way ANOVA with Tukey's *post hoc* test	6	F(3, 20) = 57.448	Con vs. CBD<br>Con vs. Aβ<br>Con vs. Aβ + CBD<br>Aβ vs. Aβ + CBD	P = 0.124<br>P < 0.001<br>P < 0.001<br>P = 0.02
Fig. 4 GPR34	One-way ANOVA with Tukey's *post hoc* test	6	F(3, 20) = 64.956	Con vs. CBD<br>Con vs. Aβ<br>Con vs. Aβ + CBD<br>Aβ vs. Aβ + CBD	P = 0.052<br>P < 0.001<br>P < 0.001<br>P = 0.002
Fig. 4 CR3	One-way ANOVA with Tukey's *post hoc* test	6	F(3, 20) = 2.018	Con vs. CBD<br>Con vs. Aβ<br>Con vs. Aβ + CBD<br>Aβ vs. Aβ + CBD	P = 0.332<br>P = 0.267<br>P = 0.04<br>P = 0.872

Fig.4 P2Y6	One-way ANOVA with Tukey's *post hoc* test	6	F(3, 20) = 5.618	Con vs. CBD<br>Con vs. Aβ<br>Con vs. Aβ + CBD<br>Aβ vs. Aβ + CBD	P = 0.032<br>P = 0.042<br>P = 0.014<br>P = 0.027
Fig. 5 TRPV 2	One-way ANOVA with Tukey's *post hoc* test	4	F(3, 8) = 56.861	WT + Aβ vs. WT + Aβ + CBD<br>WT + Aβ vs. siRNA + Aβ<br>WT + Aβ vs. siRNA + Aβ + CBD<br>WT + Aβ + CBD vs. siRNA + CBD + Aβ<br>siRNA + Aβ vs. siRNA + Aβ + CBD	P < 0.001<br>P = 0.001<br>P = 0.435<br>P < 0.001<br>P = 0.042
Fig. 5 Trem2	One-way ANOVA with Tukey's *post hoc* test	4	F(3, 8) = 53.818	WT + Aβ vs. WT + Aβ + CBD<br>WT + Aβ vs. siRNA + Aβ<br>WT + Aβ vs. siRNA + Aβ + CBD<br>WT + Aβ + CBD vs. siRNA+ CBD + Aβ<br>siRNA + Aβ vs. siRNA + Aβ + CBD	P < 0.001<br>P = 0.001<br>P = 0.014<br>P < 0.001<br>P = 0.018
Fig. 5 P2Y6	One-way ANOVA with Tukey's *post hoc* test	4	F(3, 8) = 12.986	WT + Aβ vs. WT + Aβ + CBD<br>WT + Aβ vs. siRNA + Aβ<br>WT + Aβ vs. siRNA + Aβ + CBD<br>WT + Aβ + CBD vs. siRNA+ CBD + Aβ<br>siRNA + Aβ vs. siRNA + Aβ + CBD	P = 0.038<br>P = 0.003<br>P = 0.011<br>P = 0.826<br>P = 0.014
Fig. 5 Iba1	One-way ANOVA with Tukey's *post hoc* test	4	F(3, 8) = 193.709	WT + Aβ vs. WT + Aβ + CBD<br>WT + Aβ vs. siRNA + Aβ<br>WT + Aβ vs. siRNA + Aβ + CBD<br>WT + Aβ + CBD vs. siRNA+ CBD + Aβ<br>siRNA + Aβ vs.	P < 0.001<br>P < 0.001<br>P < 0.001<br>P = 0.013<br>P = 0.008

				siRNA + Aβ + CBD	
Fig. 5 IL-4	One-way ANOVA with Tukey's *post hoc* test	4	F(3, 8) = 12.221	WT + Aβ vs. WT + Aβ + CBD<br>WT + Aβ vs. siRNA + Aβ<br>WT + Aβ vs. siRNA + Aβ + CBD<br>WT + Aβ + CBD vs. siRNA+ CBD + Aβ<br>siRNA + Aβ vs. siRNA + Aβ + CBD	*P* = 0.028<br>*P* = 0.013<br>*P* = 0.031<br>*P* = 0.467<br>*P* = 0.039
Fig. 5 GPR34	One-way ANOVA with Tukey's *post hoc* test	4	F(3, 8) = 45.404	WT + Aβ vs. WT + Aβ + CBD<br>WT + Aβ vs. siRNA + Aβ<br>WT + Aβ vs. siRNA + Aβ + CBD<br>WT + Aβ + CBD vs. siRNA + CBD + Aβ<br>siRNA + Aβ vs. siRNA + Aβ + CBD	*P* < 0.001<br>*P* = 0.008<br>*P* = 0.411<br>*P* < 0.001<br>*P* = 0.014
Fig.5 CR3	One-way ANOVA with Tukey's *post hoc* test	4	F(3, 8) = 0.721	WT + Aβ vs. WT + Aβ + CBD<br>WT + Aβ vs. siRNA + Aβ<br>WT + Aβ vs. siRNA + Aβ + CBD<br>WT + Aβ + CBD vs. siRNA+ CBD + Aβ<br>siRNA + Aβ vs. siRNA + Aβ + CBD	*P* = 0.032<br>*P* = 0.976<br>*P* = 1.00<br>*P* = 0.026<br>*P* = 1.00
Fig. 6 TRPV2	One-way ANOVA with Tukey's *post hoc* test	4	F(3, 8) = 49.106	WT + Aβ vs. WT + Aβ + CBD<br>WT + Aβ vs. siRNA + Aβ<br>WT + Aβ vs. siRNA + Aβ + CBD<br>WT + Aβ + CBD vs. siRNA+ CBD + Aβ<br>siRNA + Aβ vs. siRNA + Aβ + CBD	*P* = 0.034<br>*P* = 0.041<br>*P* < 0.001<br>*P* < 0.001<br>*P* < 0.001
Fig. 6 Trem2	One-way ANOVA with	4	F(3, 8) = 33.831	WT + Aβ vs. WT + Aβ + CBD<br>WT + Aβ vs. siRNA	*P* = 0.001<br>*P* = 0.024

	Tukey's *post hoc* test			+ Aβ	
				WT + Aβ vs. siRNA + Aβ + CBD	$P = 0.674$
				WT + Aβ + CBD vs. siRNA+ CBD + Aβ	$P = 0.004$
				siRNA + Aβ vs. siRNA + Aβ + CBD	$P = 0.005$
Fig. 6 P2Y6	One-way ANOVA with Tukey's *post hoc* test	4	$F(3, 8) = 51.478$	WT + Aβ vs. WT + Aβ + CBD	$P < 0.001$
				WT + Aβ vs. siRNA + Aβ	$P < 0.001$
				WT + Aβ vs. siRNA + Aβ + CBD	$P < 0.001$
				WT + Aβ + CBD vs. siRNA+ CBD + Aβ	$P = 0.025$
				siRNA + Aβ vs. siRNA + Aβ + CBD	$P = 0.092$
Fig. 6 Iba1	One-way ANOVA with Tukey's *post hoc* test	4	$F(3, 8) = 27.53$	WT + Aβ vs. WT + Aβ + CBD	$P = 0.002$
				WT + Aβ vs. siRNA + Aβ	$P = 0.022$
				WT + Aβ vs. siRNA + Aβ + CBD	$P = 0.18$
				WT + Aβ + CBD vs. siRNA + CBD + Aβ	$P < 0.001$
				siRNA + Aβ vs. siRNA + Aβ + CBD	$P = 0.001$
Fig. 6 IL-4	One-way ANOVA with Tukey's *post hoc* test	4	$F(3, 8) = 5.118$	WT + Aβ vs. WT + Aβ + CBD	$P = 0.554$
				WT + Aβ vs. siRNA + Aβ	$P = 0.023$
				WT + Aβ vs. siRNA + Aβ + CBD	$P = 0.004$
				WT + Aβ + CBD vs. siRNA+ CBD + Aβ	$P = 0.007$
				siRNA + Aβ vs. siRNA + Aβ + CBD	$P < 0.001$
Fig. 6 GPR34	One-way ANOVA with Tukey's *post hoc* test	4	$F(3, 8) = 55.54$	WT + Aβ vs. WT + Aβ + CBD	$P < 0.001$
				WT + Aβ vs. siRNA + Aβ	$P = 0.183$
				WT + Aβ vs. siRNA + Aβ + CBD	$P = 0.732$
				WT + Aβ + CBD vs.	$P < 0.001$

				siRNA+ CBD + Aβ<br>siRNA + Aβ vs. siRNA + Aβ + CBD	*P* = 0.043
Fig. 6 CR3	One-way ANOVA with Tukey's *post hoc* test	4	F(3, 8) = 3.754	WT + Aβ vs. WT + Aβ + CBD<br>WT + Aβ vs. siRNA + Aβ<br>WT + Aβ vs. siRNA + Aβ + CBD<br>WT + Aβ + CBD vs. siRNA + CBD + Aβ<br>siRNA + Aβ vs. siRNA + Aβ + CBD	*P* = 0.814<br>*P* = 0.025<br>*P* = 0.045<br>*P* = 0.309<br>*P* = 0.331
Fig. 7A TRPV2	One-way ANOVA with Tukey's *post hoc* test	4	F(5, 18) = 37.405	0h vs. 1h<br>0h vs. 3h<br>0h vs. 6h<br>0h vs. 16h<br>0h vs. 24h	*P* = 0.033<br>*P* = 0.026<br>*P* = 0.004<br>*P* < 0.001<br>*P* = 0.007
Fig. 7A pAkt/Akt	One-way ANOVA with Tukey's *post hoc* test	4	F(5, 18) = 26.784	0h vs. 1h<br>0h vs. 3h<br>0h vs. 6h<br>0h vs. 16h<br>0h vs. 24h	*P* = 0.016<br>*P* = 1.00<br>*P* = 0.002<br>*P* < 0.001<br>*P* = 0.001
Fig. 7A LC3-B/LC3-A	One-way ANOVA with Tukey's *post hoc* test	4	F(5, 18) = 18.745	0h vs. 1h<br>0h vs. 3h<br>0h vs. 6h<br>0h vs. 16h<br>0h vs. 24h	*P* = 0.038<br>*P* = 0.008<br>*P* = 0.034<br>*P* = 0.004<br>*P* < 0.001
Fig. 7A P62	One-way ANOVA with Tukey's *post hoc* test	4	F(5, 18) = 12.929	0h vs. 1h<br>0h vs. 3h<br>0h vs. 6h<br>0h vs. 16h<br>0h vs. 24h	*P* = 0.969<br>*P* = 0.01<br>*P* = 0.887<br>*P* = 0.031<br>*P* = 0.01
Fig. 7A Beclin e-1	One-way ANOVA with Tukey's *post hoc* test	4	F(5, 18) = 17.626	0h vs. 1h<br>0h vs. 3h<br>0h vs. 6h<br>0h vs. 16h<br>0h vs. 24h	*P* = 0.036<br>*P* = 0.028<br>*P* = 0.006<br>*P* = 0.029<br>*P* = 0.031
Fig.	One-way	4	F(2, 9) = 7.59	Con vs. Aβ + CBD	*P* = 0.028

7B TRPV 2	ANOVA with Tukey's *post hoc* test			Aβ + CBD vs. Aβ + CBD + Tra	$P = 0.047$
Fig. 7B pAkt/ Akt	One-way ANOVA with Tukey's *post hoc* test	4	$F(2, 9) = 11.461$	Con vs. Aβ + CBD<br>Aβ + CBD vs. Aβ + CBD + Tra	$P = 0.035$<br>$P = 0.011$
Fig. 7B LC3B/ LC3A	One-way ANOVA with Tukey's *post hoc* test	4	$F(2, 9) = 5.471$	Con vs. Aβ + CBD<br>Aβ + CBD vs. Aβ + CBD + Tra	$P = 0.018$<br>$P = 0.069$
Fig. 7B P62	One-way ANOVA with Tukey's *post hoc* test	4	$F(2, 9) = 12.617$	Con vs. Aβ + CBD<br>Aβ + CBD vs. Aβ + CBD + Tra	$P = 0.024$<br>$P = 0.018$
Fig. 7B Beclin e-1	One-way ANOVA with Tukey's *post hoc* test	4	$F(2, 9) = 14.512$	Con vs. Aβ + CBD<br>Aβ + CBD vs. Aβ + CBD + Tra	$P = 0.021$<br>$P = 0.012$
Fig.8 A	One-way ANOVA with Tukey's *post hoc* test	6	$F(3, 20) = 1612.792$	Con vs. CBD<br>Con vs. Aβ<br>Con vs. Aβ + CBD<br>CBD vs. Aβ + CBD<br>Aβ vs. Aβ + CBD	$P = 0.001$<br>$P < 0.001$<br>$P < 0.001$<br>$P < 0.001$<br>$P < 0.001$
Fig.8 B	One-way ANOVA with Tukey's *post hoc* test	6	$F(3, 20) = 42.464$	WT + Aβ vs. WT + Aβ + CBD<br>WT + Aβ vs. siRNA + Aβ<br>WT + Aβ vs. siRNA + Aβ + CBD<br>WT + Aβ + CBD vs. siRNA + CBD + Aβ<br>siRNA + Aβ vs. siRNA + Aβ + CBD	$P < 0.001$<br>$P = 0.033$<br>$P = 0.004$<br>$P = 0.006$<br>$P = 0.003$
Fig.8 D	One-way ANOVA with Tukey's	4	$F(3, 8) = 128.122$	Con vs. CBD<br>Con vs. Aβ<br>Con vs. Aβ + CBD<br>CBD vs. Aβ + CBD	$P = 0.002$<br>$P < 0.001$<br>$P < 0.001$<br>$P = 0.01$

	post hoc test			Aβ vs. Aβ + CBD	$P = 0.031$
Fig.8E	One-way ANOVA with Tukey's *post hoc* test	12	$F(4, 45) = 45.25$	Con vs. CBD<br>Con vs. Aβ<br>Con vs. Aβ + CBD<br>Con vs. Aβ + CBD +Rapa<br>Aβ vs. Aβ + CBD<br>Aβ vs. Aβ + CBD +Rapa<br>CBD vs. Aβ + CBD<br>CBD vs. Aβ + CBD +Rapa<br>Aβ + CBD vs. Aβ + CBD +Rapa	$P = 0.01$<br>$P = 0.002$<br>$P = 0.055$<br>$P < 0.001$<br>$P < 0.001$<br>$P < 0.001$<br>$P = 0.963$<br>$P = 0.002$<br>$P < 0.001$
Fig.8F	One-way ANOVA with Tukey's *post hoc* test	12	$F(4, 55) = 4.503$	Con vs. CBD<br>Con vs. Aβ<br>Con vs. Aβ + CBD<br>Con vs. Aβ + CBD +Rapa<br>Aβ vs. Aβ + CBD<br>Aβ vs. Aβ + CBD +Rapa<br>CBD vs. Aβ + CBD<br>CBD vs. Aβ + CBD +Rapa<br>Aβ + CBD vs. Aβ + CBD +Rapa	$P = 0.564$<br>$P = 0.991$<br>$P = 0.029$<br>$P = 0.012$<br>$P = 0.029$<br>$P = 0.012$<br>$P = 0.045$<br>$P = 0.036$<br>$P = 0.545$
Supplement Fig. 1	Unpaired, two-tailed student's *t* test analysis	3	$t(6) = 11.564$	Microglia vs. Neuron	$P < 0.001$
Supplement Fig. 2A	Date from AlzData web server (www.alzdata.org)	19		Con vs. AD	$P = 0.312$
Supplement Fig. 2B	Date from AlzData web server (www.alzdata.org)	19		EC: Con vs. AD<br>HP: Con vs. AD<br>TC: Con vs. AD<br>FC: Con vs. AD	$P = 0.058$<br>$P = 0.607$<br>$P = 0.218$<br>$P = 0.347$
Suppl	One-way	12	F(4, 56) =	Con vs. CBD	$P = 0.993$

ement Fig. 3A	ANOVA with Tukey's *post hoc* test		10.532	Con vs. Aβ<br>Con vs. Aβ + CBD<br>Con vs. Aβ + CBD + Tra<br>CBD vs. Aβ + CBD<br>CBD vs. Aβ + CBD + Tra<br>Aβ + CBD vs. Aβ + CBD + Tra	$P = 0.001$<br>$P = 0.002$<br>$P = 0.002$<br>$P = 0.039$<br>$P = 0.022$<br>$P = 0.497$
Supplement Fig. 3B	One-way ANOVA with Tukey's *post hoc* test	12	$F(4, 52) = 10.532$	Con vs. CBD<br>Con vs. Aβ<br>Con vs. Aβ + CBD<br>Con vs. Aβ + CBD + Tra<br>CBD vs. Aβ + CBD<br>CBD vs. Aβ + CBD + Tra<br>Aβ + CBD vs. Aβ + CBD + Tra	$P = 0.993$<br>$P = 0.001$<br>$P = 0.002$<br>$P = 0.002$<br>$P = 0.039$<br>$P = 0.022$<br>$P = 0.497$
Supplement Fig. 3B	One-way ANOVA with Tukey's *post hoc* test	6	$F(4, 24) = 0.628$	Con vs. Neg + Trem2<br>Con vs. Neg + TRPV2<br>Con vs. siRNA + Trem2<br>Con vs. siRNA + TRPV2	$P = 1.00$<br>$P = 0.999$<br>$P = 0.968$<br>$P = 0.805$
Supplement Fig. 4A	One-way ANOVA with Tukey's *post hoc* test	4	$F(2, 9) = 52.467$<br>$F(2, 9) = 38.195$	TRPV2: Con vs. Akti<br>Con vs. PDK1i<br>pAkt: Con vs. Akti<br>Con vs. PDK1i	$P = 0.001$<br>$P < 0.001$<br>$P = 0.003$<br>$P < 0.001$
Supplement Fig. 4B	Unpaired, two-tailed student's *t* test analysis	3	$t(6) = -3.458$<br>$t(6) = -2.911$	TRPV2: Con vs. IGF<br>pAkt: Con vs. IGF	$P = 0.013$<br>$P = 0.027$
Supplement Fig. 5	One-way ANOVA with Tukey's *post hoc* test	4	$F(2, 9) = 32.259$	Aβ vs. CBD<br>Aβ vs. CBD + Aβ<br>CBD vs. CBD + Aβ	$P = 0.005$<br>$P = 0.013$<br>$P = 0.001$

Supplement Fig. 6A	One-way ANOVA with Tukey's *post hoc* test	4	$F(3, 12) = 140.73$	Con vs. CBD<br>Con vs. Aβ<br>Con vs. Aβ + CBD<br>Aβ vs. Aβ + CBD	$P = 0.002$<br>$P = 0.089$<br>$P < 0.001$<br>$P < 0.001$
Supplement Fig. 6B	One-way ANOVA with Tukey's *post hoc* test	4	$F(3, 12) = 0.181$	Con vs. CBD<br>Con vs. Aβ<br>Con vs. Aβ + CBD<br>Aβ vs. Aβ + CBD	$P = 0.946$<br>$P = 0.907$<br>$P = 0.942$<br>$P = 0.999$
Supplement Fig. 6C	One-way ANOVA with Tukey's *post hoc* test	4	$F(3, 12) = 23.484$	Con vs. CBD<br>Con vs. LPS<br>Con vs. LPS + CBD<br>CBD vs. LPS + CBD<br>LPS vs. LPS + CBD	$P = 0.037$<br>$P = 0.009$<br>$P < 0.001$<br>$P < 0.001$<br>$P = 0.006$
